# Supplementary material for: Psychosocial job characteristics and mental health: Do associations differ by migrant status in an Australian working population sample?
Source: PLoS One. 2020 Nov 30;15(11):e0242906. doi: 10.1371/journal.pone.0242906 (PMC7703972; doi:10.1371/journal.pone.0242906)
Supplement: S1 Fig — (PDF) [file pone.0242906.s001.pdf]

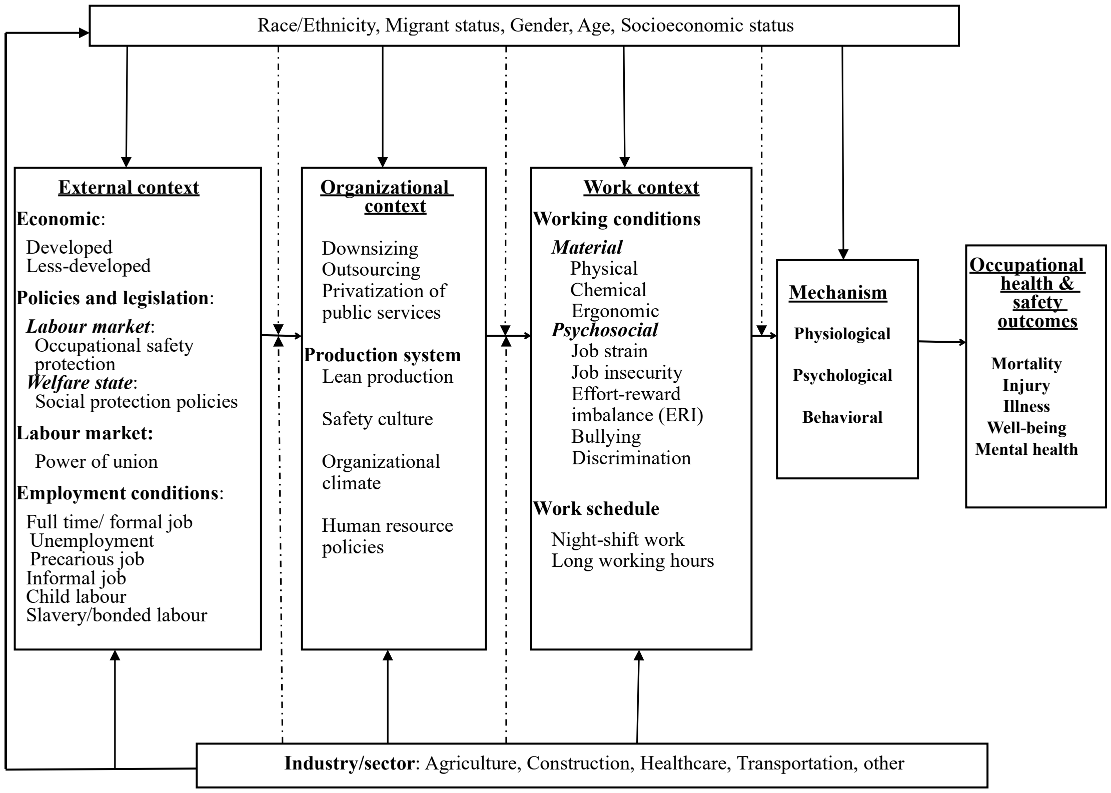

S1 Fig. Conceptual overview of the role of work organisation in the creation of OHIs (adapted and modified based on Landsbergis et al. 2014).
