# Supplementary material for: Psychosocial job characteristics and mental health: Do associations differ by migrant status in an Australian working population sample?
Source: PLoS One. 2020 Nov 30;15(11):e0242906. doi: 10.1371/journal.pone.0242906 (PMC7703972; doi:10.1371/journal.pone.0242906)
Supplement: S2 Fig — PJC: Psychosocial job characteristics; MH: Mental health; EDU: Educational attainment; Con-T: Contract type; OSL: Occupational skill level. (PDF) [file pone.0242906.s002.pdf]

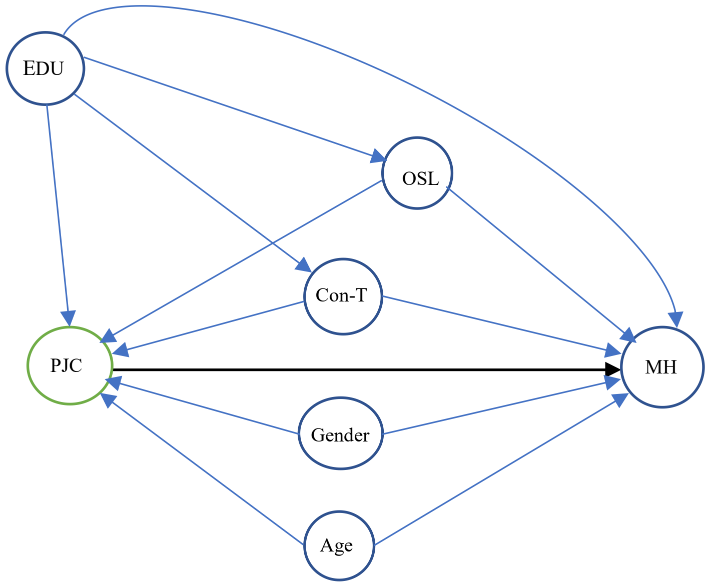

PJC: Psychosocial job characteristics; MH: Mental health; EDU: Educational attainment; Con-T: Contract type; OSL: Occupational skill level.

S2 Fig. Directed acyclic graph (DAG) of the relationship between psychosocial job characteristics and mental health.
