# Supplementary material for: Psychosocial job characteristics and mental health: Do associations differ by migrant status in an Australian working population sample?
Source: PLoS One. 2020 Nov 30;15(11):e0242906. doi: 10.1371/journal.pone.0242906 (PMC7703972; doi:10.1371/journal.pone.0242906)
Supplement: S3 Fig — Graphs generated from linear regressions with the product terms of skill discretion and migrant status and controlled for gender, age and educational attainment. Mental health was measured by the MHI-5 score. Shown with 95% CIs. (PDF) [file pone.0242906.s003.pdf]

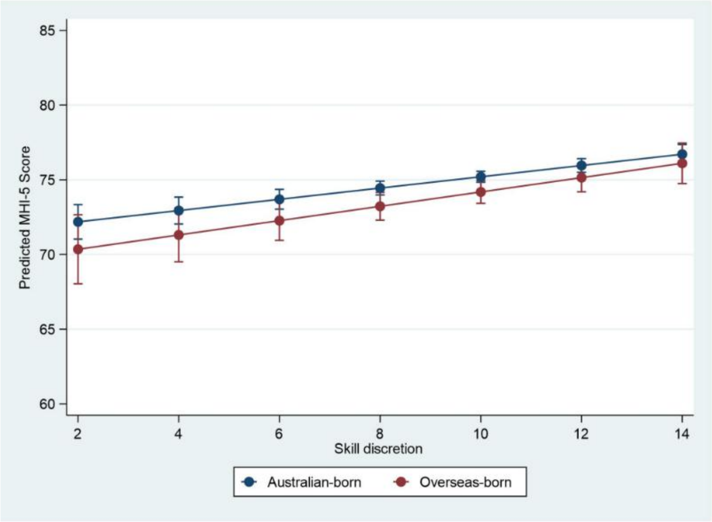

(a)

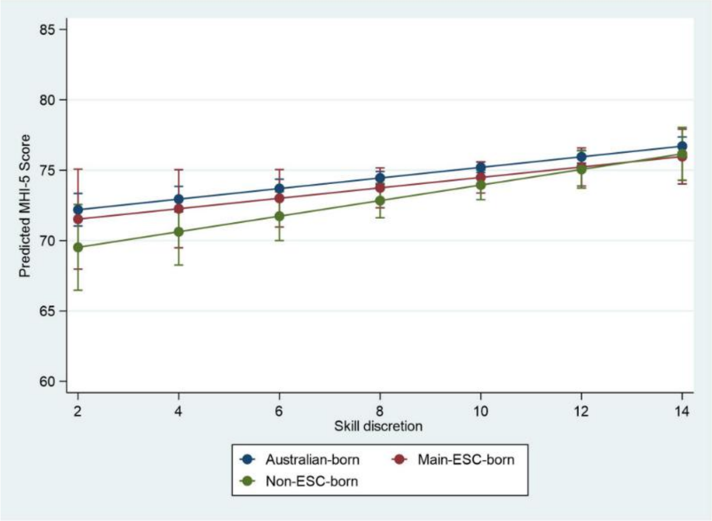

(b)

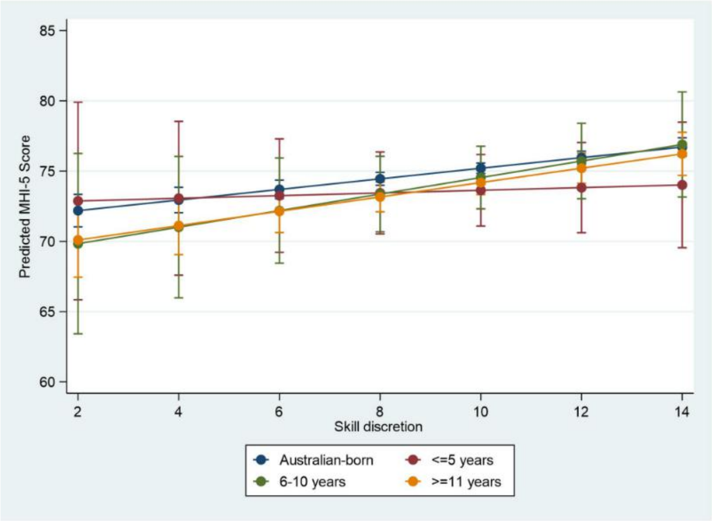

(c)

S3 Fig. Relationships between skill discretion and mental health stratified by three measures of migrant status.

Graphs generated from linear regressions with the product terms of skill discretion and migrant status and controlled for gender, age and educational attainment. Mental health was measured by the MHI-5 score. Shown with 95% CIs.
