# Supplementary material for: Psychosocial job characteristics and mental health: Do associations differ by migrant status in an Australian working population sample?
Source: PLoS One. 2020 Nov 30;15(11):e0242906. doi: 10.1371/journal.pone.0242906 (PMC7703972; doi:10.1371/journal.pone.0242906)
Supplement: S1 Table — Main-ESC-born: Born in a Main-English-speaking country, Non-ESC-born: Born in a Non-English-speaking country. (PDF) [file pone.0242906.s006.pdf]

S1 Table. Distributions of psychosocial job characteristics and MHI-5 scores by migrant status

	Migrant status	Minimum	Maximum	Median	Mean (Standard error)
**Skill discretion**	Australian-born	2	14	10	10.00 (2.76)
	Overseas-born	2	14	10	9.95 (2.73)
	Main-ESC-born†	2	14	10	10.14 (2.66)
	Non-ESC-born†	2	14	10	9.79 (2.78)
	Arrived ≤5 years	2	14	10	9.69 (2.95)
	Arrived 6-10 years	2	14	10	10.00 (2.94)
	Arrived ≥11 years	2	14	10	9.98 (2.67)
**Decision authority**	Australian-born	3	21	12	12.44 (4.66)
	Overseas-born	3	21	13	13.06 (4.57)
	Main-ESC-born†	3	21	14	13.26 (4.72)
	Non-ESC-born†	3	21	13	12.89 (4.42)
	Arrived ≤5 years	3	21	12	11.91 (3.64)
	Arrived 6-10 years	3	21	13	12.50 (4.43)
	Arrived ≥11 years	3	21	14	13.27 (4.65)
**Job insecurity**	Australian-born	3	21	8	8.47 (3.83)
	Overseas-born	3	21	9	8.91 (4.02)
	Main-ESC-born†	3	21	9	8.64 (3.89)
	Non-ESC-born†	3	21	9	9.14 (4.13)
	Arrived ≤5 years	3	21	10	10.00 (3.73)
	Arrived 6-10 years	3	21	9	9.28 (4.05)
	Arrived ≥11 years	3	21	9	8.74 (4.03)
**MHI-5 score**	Australian-born	0	100	80	75.12 (16.00)
	Overseas-born	4	100	80	74.50 (16.24)
	Main-ESC-born†	4	100	80	75.12 (16.16)
	Non-ESC-born†	8	100	76	73.96 (16.30)
	Arrived ≤5 years	24	100	76	72.86 (16.56)
	Arrived 6-10 years	16	100	80	74.04 (16.04)
	Arrived ≥11 years	4	100	80	74.74 (16.23)

† Main-ESC-born: Born in a Main-English-speaking country, Non-ESC-born: Born in a Non-English-speaking country.
