# Supplementary material for: Psychosocial job characteristics and mental health: Do associations differ by migrant status in an Australian working population sample?
Source: PLoS One. 2020 Nov 30;15(11):e0242906. doi: 10.1371/journal.pone.0242906 (PMC7703972; doi:10.1371/journal.pone.0242906)
Supplement: S2 Table — Results of univariable and multivariable linear regressions; *** p<0.001; @ Adjusted model adjusted for gender, age, educational attainment, contract type, full/part-time and occupational skill level simultaneously. Note: Controlling for contract type, full/part-time and occupational skill level in the analyses reduced the sample size to 8917 because there were 52 observations did not provide information on these three confounders. (PDF) [file pone.0242906.s007.pdf]

S2 Table. Psychosocial job characteristics and mental health: unadjusted and adjusted linear regression results of sensitivity analysis (n=8917).

	Predicted Mental Health (MHI-5 score), shown as Coefficient (95% CI)							
	Unadjusted model	Adjusted Model						
		Gender	Age	Education	Contract type	Full/part-time	Occupational skill level	Fully
**Skill discretion**	0.40 (0.28, 0.52) ***	0.40 (0.28, 0.52) ***	0.40 (0.28, 0.52) ***	0.41 (0.29, 0.53) ***	0.35 (0.23, 0.48) ***	0.35 (0.22, 0.47) ***	0.41 (0.28, 0.54) ***	0.38 (0.25, 0.51) ***
**Decision authority**	0.41 (0.34, 0.48) ***	0.39 (0.32, 0.46) ***	0.39 (0.31, 0.46) ***	0.41 (0.34, 0.49) ***	0.42 (0.34, 0.49) ***	0.39 (0.32, 0.46) ***	0.41 (0.34, 0.49) ***	0.40 (0.32, 0.48) ***
**Job insecurity**	-1.04 (-1.13, -0.96) ***	-1.07 (-1.15, -0.98) ***	-1.05 (-1.13, -0.97) ***	-1.05 (-1.13, -0.96) ***	-1.04 (-1.12, -0.96) ***	-1.04 (-1.12, -0.95) ***	-1.05 (-1.13, -0.96) ***	-1.08 (-1.17, -1.00) ***

Results of univariable and multivariable linear regressions; *** p<0.001; @ Fully adjusted model adjusted for gender, age, educational attainment, contract type, full/part-time and occupational skill level simultaneously.

Note: Controlling for contract type, full/part-time and occupational skill level in the analyses reduced the sample size to 8917 because there were 52 observations did not provide information on these three confounders.
