# Supplementary material for: Psychosocial job characteristics and mental health: Do associations differ by migrant status in an Australian working population sample?
Source: PLoS One. 2020 Nov 30;15(11):e0242906. doi: 10.1371/journal.pone.0242906 (PMC7703972; doi:10.1371/journal.pone.0242906)
Supplement: Attachment — Submitted filename: Response to Reviewers.docx [file pone.0242906.s008.docx]

**Academic editor**

**Major concerns**

**AE-1. It should be addressed why it is important to study migrant status as a boundary condition of the relationship between job stressors and strains. This should go along with a clear definition of job stressors and strains. Furthermore, it is important to distinguish between job stressors (e.g. job insecurity), job resources (e.g. skill discretion, decision authority, social support), and strains (e.g. deteriorated mental health). A low level of a job resource does not necessarily imply that a stressor is present. The manuscript needs to be repositioned as skill discretion and decision authority do not represent job stressors. I see two different ways to deal with this issue: On the one hand, it is possible to look at migrant status as the modifier of the relationships between job stressors and strain as well job resources and strain. On the other hand, HILDA might include other variables that are commonly understood as job stressors (e.g. workload, social conflict).**

**Response:**

Thank you for the comments. We recognize that there is diversity in the theoretical models relating to psychosocial work environments and health. We have adopted the Job Demand-Control (JDC) model in this study. In the JDC model, psychosocial job characteristics are treated as independent exposures, thus we treat skill discretion, decision authority and job insecurity as exposures in our study. We agree with you that the term ‘job stressors’ may result in confusion, and have changed it to ‘psychosocial job characteristics’ in this revised manuscript. Furthermore, we understand that ‘strain’ can be used to define exposure-associated ill-mental health; however, ‘strain’ as used in the term ‘job strain’ refers to the combination of high job demands and low job control in the JDC model; thus, we prefer to not use ‘strain’ in this context to avoid confusion. Moreover, as per your suggestion, we have revised our introduction to provide a clearer definition of occupational health inequalities (OHIs) and the importance of migrant status in research on OHIs.

***The corresponding revisions to the text are:***

***Title***

*Do psychosocial job characteristic-associated impacts on mental health differ by migrant status? An Australian comparative analysis*

***Introduction (page 3-4, line 52-78)***

*Occupational health inequalities (OHIs) refer to avoidable differences in occupational exposures or work-related health outcomes between different occupational population groups [1, 2]. Disadvantaged population groups, such as workers with lower socioeconomic status, females and migrant workers, may be more likely to experience OHIs [1, 3]. According to an OHI framework developed by Landsbergis et al. (1) (see supporting information* ***S1 Fig****), this may be due to disadvantaged population groups having higher risks of adverse occupational exposures, or they may be more vulnerable to exposure-associated health impacts.*

*Migrant workers are more likely to be engaged in low-skilled and so-called ‘3D’ jobs (dirty, dangerous and demeaning), which often come with high exposures to occupational hazards, including physical, chemical, and biological factors, as well as adverse psychosocial job characteristics [4, 5]. Furthermore, migrant workers may have a higher vulnerability to adverse occupational exposures due to many disadvantages, such as language difficulty, culture shock, lack of social and family support and lack of access to health care. These may result in migrant workers having higher risks of work-related accidents, injuries, fatalities, physical and mental health problems than native-born workers [6, 7]. Hence, migrant workers, in general, are characterised as more likely to experience OHIs [1, 3, 8].*

*Mental health problems are common among working-age populations and result in a heavy health burden [9, 10]. While work is generally considered to be good for mental health, poor working conditions―including poor psychosocial work environments can result in mental health problems or may worsen existing problems [9].* *Psychosocial job characteristics refer to ‘factors involved with psychosocial processes linked to the social environment of work that may be important in the causation of illness’ [11]; these are common exposures to which all workers may potentially be exposed. Adverse psychosocial job characteristics―including high job demands, low job control, job strain, job insecurity, effort-reward imbalance and lack of social support at work―have been shown to be associated with a wide range of adverse mental health outcomes,* *such as depression, anxiety, burnout, low life satisfaction, and suicidality and suicide mortality [12-16].*

**AE-2. The study should be presented within a theoretical framework implying that other socio-demographic differences could be either considered in the data analysis to be ruled out as potential confounders or discussed (see Reviewer 1, major concern).**

**Response:**

Thank you for this comment. We have addressed this comment in the response to Reviewer 1 (below).

**AE-3. The conduction (see Reviewer 2, major concern) and presentation (see Reviewer 1, minor concern) of the data analysis should be revised.**

**Response:**

Thank you for this comment. We have addressed these comments in the response to Reviewers 1 and 2, respectively.

**AE-4. More attention to detail throughout the manuscript would be warranted (see several concerns raised by Reviewer 2).**

**Response:**

Thank you for this comment. We have edited and proofread the manuscript carefully.

**Reviewer 1**

This is an interesting article on a relevant issue for occupational health / public health that focus on a topic of growing interest. I want to congratulate the Authors for the ideas, effort and quality of their work. After reading the article in deep, I have some major and minor comments. Hopefully they will be useful to the Authors.

**Major comments**

**R1-1. For me, there is a surprising thing in this article: the absence of some key concepts (and variables) theoretically related in the literature to psychosocial exposures, mental health and migrant status: 1) “social class” (or any proxi as “occupational grade” or “socioeconomic status”..., i.e. “manual / non manual workers”, “blue / white collar”, ESEC categories..., depending on the available data and the theoretical background); and 2) employment conditions, as type of contract, i.e. fixed or temporary, partial time or full time work, level of precariousness... All of them have been related to psychosocial exposures, mental health and migrant status in other studies; but only low occupational position is mentioned once in the introduction with no consequences in the analysis and discussion. The Authors find almost no differences on psychosocial exposures regarding migrant status; perhaps because what would explain main differences could be social class / occupational grade or employment conditions?
Even considering that the unemployed at the time of interview were excluded in this study, the study population may still be a mixture of persons employed under very different conditions that are related to exposures and outcomes, mainly if the work is temporary or permanent, and full-time or part-time; low or high grade. The Authors mention that Australian-born workers have a much lower proportion (12 vs 25%) of workers with a postgraduate degree than migrants Non-ESC-born, but no differences in occupational class / category /grade; temporary / permanent, full / partial? I wonder if HILDA does not include any variable that could be used at least as a proxy of these constructs to take them into account in the analysis as a potential confounders, since they may relate to both exposures and outcome; but I think these are two important issues that should be addressed in the paper, and at least mentioned in the introduction and considered in the discussion–if no possible in the analysis**

**Response:**

Thank you for the comments.

We agree that many factors―not only socio-demographic but also employment-related―potentially confound the relationship between psychosocial job characteristics and mental health. We acknowledge this, and tested contract type, full/part-time and occupational skill level as potential confounders when we wrote the previous manuscript. However, these variables did not greatly impact on the beta coefficients in the models, and we have presented the most parsimonious models as our main results. Furthermore, it is arguable whether all such ‘potential confounders’ should be included in these models. Contract type and occupational skill level may be potential mediators of associations between educational attainment and psychosocial job characteristics, as well as mental health. To clarify this issue, we have developed and included a directed acyclic graph (DAG), and included it as supporting information **S2 Fig** in the revised manuscript. Thus, we would argue that controlling for contract type and occupational skill level in addition to educational attainment may result in an over-adjustment. Further, full/part-time overlaps with working hours and might be best operationalised as a work characteristic.

Nevertheless, we have added sensitivity analyses to show results after controlling for these employment-related factors―including contract type, full/part-time and occupational skill level (see supporting information **S1 Table**), and explained why we did not include them in the main analysis in the Results section.

Finally, we have used the most parsimonious model because interactions were tested in our study, and unnecessary covariates would further reduce statistical power (interaction tests are often under-powered).

***The corresponding revisions to the text are:***

***Sensitivity analysis (page 11, line 231-247)***

*Some employment-related factors―including contract type, full/part-time and occupational skill level―were potential confounders of the relationship between psychosocial job characteristics and mental health [17-19]. However, contract type and occupational skill level may be potential mediators of associations between educational attainment and psychosocial job characteristics, as well as mental health (see supporting information S2 Fig); thus, controlling them in addition to educational attainment may result in an over-adjustment. Furthermore, full/part-time overlaps with working hours and might be better treated as a job characteristic. We would like to include a minimal number of confounders because interactions were tested in this study―which is often under-powered, and unnecessary covariates would further reduce the statistical power. Therefore, we conducted sensitivity analyses to test whether these employment-related factors should be controlled as confounders by including them one by one into linear regressions, and then, simultaneously with gender, age and educational attainment (see supporting information* ***S1 Table****).*

*Contract type was* *a four-category variable: permanent contract, casual employment, fix-term contract and self-employment. Full-time workers* *were those who work ≥35 hours per week. Occupational skill level included four categories: highest skill, mid-high skill, mid-low skill and lowest skill [20].*

***Results (page 13-14, line 291-294)***

*Based on the results of sensitivity analysis (see supporting information* ***S1 Table****), including contract type, full/part-time and occupational skill level, either separately or simultaneously, into the linear regressions only resulted in small changes in the coefficients; therefore, they were excluded from the main analysis in this study.*

***S1 Table. Psychosocial job characteristics and mental health: Unadjusted and adjusted linear regression results (n=8917).***

|  | | ***Skill discretion*** | ***Decision authority*** | ***Job insecurity*** |
| --- | --- | --- | --- | --- |
| ***Unadjusted*** | *Beta coefficient (95% CI)* | *0.40 (0.28, 0.52) ^***^* | *0.41 (0.34, 0.48) ^***^* | *-1.04 (-1.13, -0.96) ^***^* |
|  | *Constant* | *70.96* | *69.84* | *83.91* |
| ***Adjusted for gender*** | *Beta coefficient (95% CI)* | *0.40 (0.28, 0.52) ^***^* | *0.39 (0.32, 0.46) ^***^* | *-1.07 (-1.15, -0.98) ^***^* |
|  | *Constant* | *72.20* | *71.14* | *85.57* |
| ***Adjusted for age*** | *Beta coefficient (95% CI)* | *0.40 (0.28, 0.52) ^***^* | *0.39 (0.31, 0.46) ^***^* | *-1.05 (-1.13, -0.97) ^***^* |
|  | *Constant* | *69.70* | *69.31* | *82.30* |
| ***Adjusted for educational attainment*** | *Beta coefficient (95% CI)* | *0.41 (0.29, 0.53) ^***^* | *0.41 (0.34, 0.49) ^***^* | *-1.05 (-1.13, -0.96) ^***^* |
|  | *Constant* | *70.87* | *69.80* | *83.51* |
| ***Adjusted for contract type*** | *Beta coefficient (95% CI)* | *0.35 (0.23, 0.48) ^***^* | *0.42 (0.34, 0.49) ^***^* | *-1.04 (-1.12, -0.96) ^***^* |
|  | *Constant* | *71.77* | *70.29* | *83.80* |
| ***Adjusted for full/part-time*** | *Beta coefficient (95% CI)* | *0.35 (0.22, 0.47) ^***^* | *0.39 (0.32, 0.46) ^***^* | *-1.04 (-1.12, -0.95) ^***^* |
|  | *Constant* | *72.10* | *70.63* | *84.48* |
| ***Adjusted for occupational skill level*** | *Beta coefficient (95% CI)* | *0.41 (0.28, 0.54) ^***^* | *0.41 (0.34, 0.49) ^***^* | *-1.05 (-1.13, -0.96) ^***^* |
|  | *Constant* | *71.51* | *70.36* | *84.51* |
| ***Adjusted^﹫^*** | *Beta coefficient (95% CI)* | *0.38 (0.25, 0.51) ^***^* | *0.40 (0.32, 0.48) ^***^* | *-1.08 (-1.17, -1.00) ^***^* |
|  | *Constant* | *71.81* | *71.16* | *83.85* |

*Results of univariable and multivariable linear regressions; Mental health was measured by MHI-5 score; *** p<0.001; ﹫Adjusted model adjusted for gender, age, educational attainment, contract type, full/part-time and occupational skill level simultaneously*

**Minor comments**

**R1-2. In my opinion, data source section of the article is quite difficult to read, with many population numbers given that may confuse the reader on the final sample size.**

**Response:**

Thank you for the comment. We have revised this paragraph.

***The corresponding revisions to the text are (page 6, line 123-133):***

*The data come from the Household Income and Labour Dynamics in Australia Survey (HILDA). HILDA is a nationally representative sample of Australian households, whose data collection began in 2001 and is conducted annually [21]. It covers a wide range of information on family life, household composition, income, labour market activity, employment, socioeconomic status and health. All household members aged 15 and older were interviewed through face-to-face or telephone interviews. In wave 1 (2001), 13969 persons responded to the survey, and the response rate was 92.3%. In 2011, an additional 4280 persons were added to top up the sample size to allow a better representation of the Australian population. Wave 14 (2014-2015) was used in the current study. In this wave, 17325 individuals responded to the survey, and the response rate for the parent sample was 80.8% [22]. The final analytic sample consists of 8969 respondents. More information on the analytic sample for this paper is described under ‘Inclusion and exclusion criteria’ below.*

**R1-3. What is the final response rate?**

**Response:**

The final response for the parent sample rate was 80.8%. We have reported it in the Materials and methods section now. Please see the response to your previous comment.

**R1-4. I would suggest edit Table 1, making it clear that columns 3 to 7 refer only to overseas-born workers.**

**Response:**

Thank you for the comment. We have revised **Table 1** accordingly.

***The corresponding revisions to the text are (page 12, line266-267):***

***Table 1. Descriptive statistics on socio-demographic characteristics by migrant status (n=8969).***

|  | *Australian-born, n (%)* | *Migrant workers* | | | | | |
| --- | --- | --- | --- | --- | --- | --- | --- |
|  |  | ***Overseas-born, n (%)*** | ***Main-ESC-born^†^, n (%)*** | ***Non-ESC-born^†^, n (%)*** | ***Arrived ≤5 years, n (%)*** | ***Arrived 6-10 years, n (%)*** | ***Arrived ≥11 years, n (%)*** |
| *Overall* | *7238 (80.70)* | *1731 (19.30)* | *804 (8.96)* | *927 (10.33)* | *154 (1.72)* | *199 (2.22)* | *1378 (15.36)* |
| *Gender* |  |  |  |  |  |  |  |
| *Male* | *3626 (50.10)* | *919 (53.09)* | *461 (57.34)* | *458 (49.41)* | *76 (49.35)* | *111 (55.78)* | *732 (53.12)* |
| *Female* | *3612 (49.90)* | *812 (46.91)* | *343 (42.66)* | *469 (50.59)* | *78 (50.65)* | *88 (44.22)* | *646 (46.88)* |
| *Age (years)* |  |  |  |  |  |  |  |
| *15-24* | *1486 (20.53)* | *109 (6.30)* | *45 (5.60)* | *64 (6.90)* | *29 (18.83)* | *25 (12.56)* | *55 (3.99)* |
| *25-34* | *1662 (22.96)* | *370 (21.37)* | *134 (16.67)* | *236 (25.46)* | *84 (54.55)* | *81 (40.70)* | *205 (14.88)* |
| *35-44* | *1498 (20.70)* | *416 (24.03)* | *183 (22.76)* | *233 (25.13)* | *29 (18.83)* | *64 (32.16)* | *323 (23.44)* |
| *45-54* | *1582 (21.86)* | *493 (28.48)* | *269 (33.46)* | *224 (24.16)* | *8 (5.19)* | *25 (12.56)* | *460 (33.38)* |
| *55-64* | *1010 (13.95)* | *343 (19.82)* | *173 (21.52)* | *170 (18.34)* | *4 (2.60)* | *4 (2.01)* | *335 (24.31)* |
| *Educational attainment* |  |  |  |  |  |  |  |
| *High school or lower* | *2621 (36.21)* | *398 (22.99)* | *218 (27.11)* | *180 (19.42)* | *28 (18.18)* | *38 (19.10)* | *332 (24.09)* |
| *Diploma or certificate* | *2530 (34.95)* | *553 (31.95)* | *302 (37.56)* | *251 (27.08)* | *38 (24.68)* | *44 (22.11)* | *471 (34.18)* |
| *Bachelor* | *1232 (17.02)* | *405 (23.40)* | *142 (17.66)* | *263 (28.37)* | *50 (32.47)* | *57 (28.64)* | *298 (21.63)* |
| *Postgraduate* | *855 (11.81)* | *375 (21.66)* | *142 (17.66)* | *233 (25.13)* | *38 (24.68)* | *60 (30.15)* | *277 (20.10)* |

*† Main-ESC-born: Born in a Main-English-speaking country; Non-ESC-born: Born in a Non-English-speaking country*

**Reviewer 2**

This manuscript assesses whether migrant workers are more vulnerable to job stressor exposures in terms of mental health, based on a survey in 2014-2015 among 8970 persons in an Australian working population-representative sample. Few previous studies have examined migrant status-based differences in job stressor—mental health associations. Therefore, whether migrants are differentially affected by job stressor exposures is unclear, especially when language proficiency and years since arrival in host country are taken into account. Thus, this topic is relevant and interesting.

**Major comments**

**R2-1. The manuscript is mostly well-written. However I have some comments. The authors conclude that they found little evidence that the relationship between job stressor exposures and mental health is modified by migrant status. However, the stratified analyses, presented in the figures, show that migrants who arrived ≤5 years previously differed from other groups. Also migrants born in a Non-English speaking country seem to differ somewhat from other groups. The stratified analyses, thus, indicate that there is effect modification. This is my major comment.**

**Response**

Thank you for the comments. In this study, the effect-measure modification was investigated by fitting interaction terms and we followed standard statistical procedures to determine whether there was potential effect-measure modification [23], and have now expanded our discussion of this below and in the manuscript.

Firstly, we tested whether the potential interaction/effect-measure modification was statistically significant using the Likelihood ratio (LR) test. This involved fitting a fully adjusted model-1 with variables A and B (A+B) and then a second fully adjusted model-2 with an interaction term for A and B (A + B + AxB). The LR test determined whether the interaction/effect-measure modification of A and B was statistically significant by a p-value. However, the p-value only provides a measure of statistical significance and no information on the magnitude of associations. Therefore, it was followed by a second step―graphing the potential effect-measure modification with 95% confidence intervals (CI) to visualize the magnitude of the association and see if there was any indication of possible effect-measure modification. The third step would be stratifying the association between exposure and outcome by the effect-measure modifier if the LR test result had a p-value less than 0.05.

In some of our analyses, the LR test resulted in a large p-value―indicating no statistical evidence of effect-measure modification, while graphs showed some suggestion of effect-measure modification. This could be due to the small sample size of migrants who arrived ≤5 years previously (n=154), thus limiting statistical power. That is, the small subgroup may have a wide 95% CI and, as a result, some 95% CIs were overlapped in our models and, thus, were consistent with the LR test results giving p-values far great than 0.05. We have revised the graphs of the psychosocial job characteristic―mental health associations by migrant status with 95% CIs (see **Figs 2 and 3**). It can be seen that the 95% CI of migrants who arrived ≤5 years largely overlapped with other subgroups even though the slopes of this subgroup looked different from that of other groups. This explains our reporting that there is in some instances a suggestion of effect-measure modification, though a larger study with higher power would be required to further investigate this.

We have revised the Materials and methods, Results, and Discussion sections to explain this clearly and have now included the subgroup 95% CI for each plotted line in our graphs.

***The corresponding revisions to the text are:***

***Materials and methods (Page 10, line 220-228)***

*Third, Likelihood ratio (LR) tests were used to assess whether the potential effect-measure modification by migrant status of psychosocial job characteristic―mental health relationships were statistically significant. Considering that LR tests only provided measures of statistical significance without information on the relative magnitude of associations, they were complemented for descriptive purposes by graphing the relationships between psychosocial job characteristics and mental health by migrant status with 95% confidence intervals (CIs) to visualise the magnitude of associations and see if there was any indication of possible effect-measure modification. If the LR test results were significant (p<0.05), the relationships between psychosocial job characteristics and mental health would be stratified by the effect modifiers.*

***Results (Page 14, line 305-309)***

*When graphing the effect-measure modification results, we found that in both skill discretion―mental health (see* ***Fig 2****) and decision authority―mental health (see* ***Fig 3****) relationships, the regression lines for migrants who arrived ≤5 years previously appeared to differ from the lines for other groups. The slopes of the regression lines for migrants who arrived ≤5 years previously were close to zero; however, their 95% CIs largely overlapped with that of other groups.*

***Fig 2.*** *Relationships between skill discretion and mental health stratified by migrant status.*

*Graphs generated from linear regressions with the interaction terms of skill discretion and migrant status and controlled for gender, age and educational attainment. Mental health was measured by MHI-5 score. Shown with 95% CIs.*

***Fig 3.*** *Relationships between decision authority and mental health stratified by migrant status.*

*Graphs generated from linear regressions with the interaction terms of decision authority and migrant status and controlled for gender, age and educational attainment. Mental health was measured by MHI-5 score. Shown with 95% CIs.*

***Discussion***

***(Page 15-16, line 334-342)***

*Inconsistent with our hypothesis, the magnitude of associations between psychosocial job characteristics and mental health did not vary by migrant status in our working Australian population sample despite investigating migrant status with three distinct measures. However, there was a suggestion that psychosocial job characteristic―mental health associations for migrants who had arrived in Australia ≤5 years previously differed from the associations for migrants who had been living in Australia for longer, though we could not rule out the possibility that this difference arose by chance. Skill discretion and decision authority were positively associated with MHI-5 score for both Australian-born workers and most subgroups of migrants, but there were non-statistically significant suggestions that regression lines were fairly flat for the most recently arrived migrants.*

***(Page 16-17, line 358-364)***

*We do, however, find suggestive, non-statistically significant evidence that the most recently arrived migrants may not be sensitive to skill discretion and decision authority, suggesting associations for these two psychosocial job characteristics are stronger in Australian-born workers (see* ***Figs 2*** *and* ***3****); however, these apparent differences may be due to the small sample size of the subgroup of migrants who arrived in Australia ≤5 years previously (n=154). Future study with a larger sample size would be required to resolve this question.*

**Minor comments**

Other comments are mostly spelled out in detail for the respective sections of the manuscript.

**Title:**

**R2-2. The title of the manuscript ("vary by migrant status") does not quite correspond with the aim of the study ("more vulnerable").**

**Response:**

Thank you for the comment. This study aimed to test whether the vulnerability to psychosocial job characteristic-associated impacts on mental health varies by migrant status and we hypothesized migrant workers may be more vulnerable than native-born workers. We have revised our Title to make it clearer.

***The corresponding revision to the text is:***

***Title***

*Do psychosocial job characteristic-associated impacts on mental health differ by migrant status? An Australian comparative analysis*

**Abstract:**

**R2-3. The year of the survey would be much more informative to the reader than the wave number.**

**Response:**

Thank you for the comment. We have added the year of the survey.

***The corresponding revision to the text is:***

***(page 2, line 34-36)***

*……A total of 8969 persons from wave 14 (2014-2015) of the Household Income and Labour Dynamics in Australia Survey were included in the analysis…….*

**R2-4. In order to understand the results, the reader would need to know that higher MHI-5 scores indicate better mental health (contrary to Hopkins symptom check List, HSCL), and I suggest adding this information to the abstract.**

**Response:**

Thank you for the comment. We have added the indication of MHI-5 in the Abstract.

***The corresponding revision to the text is:***

***(page 2, line 37-38)***

*Mental health was assessed via a Mental Health Inventory-5 score (MHI-5), with a higher score indicating better mental health.*

**Introduction:**

**R2-5. Lines 98-101: The reference to this sentence (Migrant workers…) seems to be missing.**

**Response:**

Thank you for the comment. We have added references.

***The corresponding revision to the text is:***

***(page 5, line 106-109)***

*Migrant workers in Australia have been shown to experience lower skill discretion/complexity and higher job insecurity than Australian-born workers, especially those from Non-English speaking countries and recently arrived migrants [24, 25].*

**Material and methods:**

**R2-6. It is stated that the study participants were interviewed through face-to-face or telephone interviews. However, there also seems to have been a Self-Completion Questionnaire, mentioned further down.**

**Response:**

Thank you for the comment. The HILDA survey comprised four different instruments: the Household Form (HF), the Household Questionnaire (HQ), the Person Questionnaire (PQ), and the Self-Completion Questionnaire (SCQ). The vast majority of data was collected through face-to-face interviews, and a small number was collected over the telephone. However, the SCQ data was not collected by face-to-face interview or telephone because the SCQ covers some topics that may make respondents slightly uncomfortable. All persons who completed the PQ were asked to complete the SCQ, but the SCQ was left with the respondents and collected at a later date or returned in the mail. We have revised our manuscript to only discuss the SCQ in the Missing data sub-section in the hope this may avoid further confusion.

***The corresponding revisions to the text are:***

***Materials and methods (page 6, line 126-133)***

*All household members aged 15 and older were interviewed through face-to-face or telephone interviews. In wave 1 (2001), 13969 persons responded to the survey, and the response rate was 92.3%. In 2011, an additional 4280 persons were added to top up the sample size to allow a better representation of the Australian population. Wave 14 (2014-2015) was used in the current study. In this wave, 17325 individuals responded to the survey, and the response rate for the parent sample was 80.8% [22]. The final analytic sample consists of 8969 respondents. More information on the analytic sample for this paper is described under ‘Inclusion and exclusion criteria’ below.*

***Missing data (page 9, line 198-201)***

*Based on the reason for missing data provided by HILDA, 1184 among the 1586 were due to non-response to the self-completion questionnaire (SCQ) of the HILDA survey, where psychosocial job characteristics were measured.*

**R2-7. P. 6: Job control was measured by two subscales (line 140), however, further down one of the subscales (decision authority) is referred to as (job) control (line 143). These were measured from 1 to 7, and seem to be a Likert scale, but this is not stated, as for the outcome and job insecurity.**

**Response:**

We defined decision authority by ‘*Decision authority assess the opportunity for control……*’ *(Page 7, line 150)* there. We have explained all the three psychosocial job characteristic scales and MHI-5 were Likert scale in our manuscript. For skill discretion and decision authority, we said: “*All the items were scored from 1 ‘strongly disagree’ to 7 ‘strongly agree’……*” *(Page 7, line 153).* For job insecurity, we said, “*Job insecurity……, which was assessed by three items with the same 7-point Likert scale*” *(Page 7, line 159)*. For the MHI-5, we said: “*The MHI-5 consists of five six-point Likert items…… (page 6-7, line 138-139)*”.

**R2-8. P. 7: I would suggest referring to confounders and effect modifiers as POTENTIAL confounders and effect modifiers in the Methods section, before doing the analyses.**

**Response:**

Thank you for the comments. We have revised the Materials and methods section using “Potential confounders” and “Potential effect modifier”.

**R2-9. P. 8: Inclusion, exclusion and missing data: I would suggest including a flow chart showing this. In the Missing data part, it is not so clear whether the different categories of missing data are overlapping or if the numbers with missing data for each step only include those without missing data in the previous step.**

**Response:**

Thank you for the comments. We have revised the Inclusion and exclusion section and created a participant flow chart (see **Fig 1**) to make it clearer. There were 1606 missing cases (10575-8969=1606), the categories were not overlapping. It also has been shown in **Fig 1**.

***The corresponding revisions to the text are (page 9, line 189-192):***

*The parent sample consisted of 17325 individuals in wave 14. As shown in* ***Fig 1****, 15231 of them were aged 15-64 years and of these, 10575 were employed, 10569 provided information of all confounders and migrant status. Among these 10569, 8983 observations answered all the items of the three psychosocial job characteristic scales, and 8969 also had MHI-5 score and, thus, were included in the analysis.*

**Fig 1.** Participant flow into the analytic sample.

**R2-10. Lines 191-195: The different reasons for refusing to answer (or not stated?) do not seem so interesting to me (but I may be wrong).**

**Response:**

Thank you for the comments. A total of 1586 sample were excluded from our analysis due to missing items in psychosocial job characteristic scales, which may result in bias due to missing data. We described the missing data in detail to show that in most instances, all psychosocial job characteristic items were missing for all three scales; thus, possibilities for value substitution were limited. Now, as per your suggestion, we have shortened the whole paragraph on Missing data.

***The corresponding revision to the text is (page 9-10, line 197-208):***

*There were 1606 respondents, accounting for 15.2% of the employed sample excluded from our analysis―among whom, 1586 were due to missing items of psychosocial job characteristic scale. Based on the reason for missing data provided by HILDA, 1184 among the 1586 were due to non-response to the self-completion questionnaire (SCQ) of the HILDA survey, where psychosocial job characteristics were measured. Other reasons included the SCQ not being asked (n=286), multiple responses to the SCQ (n=21) and refused/not stated (n=95). Among the 95 who refused to answer, 9 were missing all items for the three psychosocial job characteristic scales. Based on these reasons, a total of 1479 (1184+286+9) were missing all items of the three psychosocial job characteristic scales; moreover, a small number were excluded due to multiple responses (n=21) and missing information of education (n=1), migrant status (n=5) and MHI-5 score (n=14). This left 86 respondents who were missing one or two items of the psychosocial job characteristic scales thus being excluded from analysis, limiting possibilities for value substitution (e.g., mean substitution of single missing items).*

**R2-11. Lines 195-197: Were the 86 respondents excluded?**

**Response:**

Yes, they were those who were excluded from the analysis due to missing one or two items in any of the psychosocial job characteristic scales.

**R2-12. P. 9, lines 200-202: As all p-values are <0.001, I suggest writing this only once, at the end of the sentence. The same comment applies to the presentation of the characteristics of subpopulations (P. 10, lines 228-233).**

**Response:**

Thank you for the comments. We have revised the paragraph according to your comments.

***The corresponding revisions to the text are:***

***Missing data (page 10, line 210-214)***

*Compared to the observations included in the analysis, the exclusions were more likely to be male (56.4% vs. 50.7%), younger (15_24 years: 24.3% vs. 17.8%), lower educated (high school or lower: 38.7% vs. 33.7%), lower in skill level (low skill level: 18.4% vs. 14.0%; high skill level: 32.6% vs. 38.7%), casual employment (23.8% vs. 19.0%) and Non-ESC-born migrants (13.8% vs. 10.3%) (all p-values <0.001).*

***Results (page 12, line 257-264)***

*The differences in gender, age and educational attainment between Australian-born workers and migrant workers were apparent (all p-values <0.001). For example, Australian-born workers included a significantly lower proportion of males than Main-ESC-born workers (50.1% vs. 57.3%). Australian-born workers included a significantly lower proportion of workers with a postgraduate degree compared to Non-ESC-born workers (11.8% vs. 25.1%), especially compared to migrants who arrived in Australia between 6 to 10 years previously (30.2%). In contrast, Australian-born workers included a significantly higher proportion of workers between 15 to 24 years of age than overseas-born workers (20.5% vs. 6.3%).*

**Statistical analysis:**

**R2-13. Linear regression was used to assess the relationships between job stressors and mental health. Did you check that these relationships actually are linear, by plotting the exposure and outcome data? If they are not, it might be more correct to treat the variables as categorical data, e.g. by combing the levels into a few categories, using the highest category (for job control) and the lowest category (for job insecurity) as reference, respectively.**

**Response:**

Yes, we have checked the linear relationships between psychosocial job characteristics and MHI-5. Though they were not strong linear relationships (as shown in the example below), we still considered them linear and, hence, decided against creating categorical variables. From an epidemiological research perspective we were interested in the effect of continuous determinants on continuous outcomes and, thus, dichotomizing/categorizing a continuous variable may lead to a loss of information and statistical power. In this study, interactions were tested―which is often under-powered, therefore, we prefer to treat psychosocial job characteristics as continuous to maximise statistical power.

Relationship between job insecurity and MHI-5

**R2-14. LR tests were used to assess migrant status as an effect modifier of the job stressor—mental health relationships. However, this may be better evaluated in stratified analyses, as commented below (Results section).**

**Response:**

Thank you for your comment. We have addressed it in our response to major comment R2-1.

**Results**

**R2-15. Some of the results of Table 1 are presented in the text of this section, without the decimals, which is perfectly fine and, I would also suggest showing maximum 1 decimal in the table.**

**Response:**

Thank you for your comments. We prefer to keep 2 decimals in the tables because our other papers kept 2 decimals and we would like to be consistent. Following your suggestion, we have revised our manuscript to present 1 decimal in the whole text.

**R2-16. A couple of places in the Results section, the authors write that the results suggest (lines 245, 275) or indicate (l. 359) something. This is an interpretation of the results and should be moved to the discussion.**

**Response:**

Thank you for your comments. We have revised the Results section and move them to the Discussion section.

***The corresponding revisions to the text are:***

***Results***

***(page 13, line 286-289)***

*……the difference in predicted MHI-5 score between the minimum and maximum job characteristic scale values were 71.8—76.6 for skill discretion, 71.1—78.5 for decision authority, and 80.8—62.1 for job insecurity. The range of predicted MHI-5 score of job insecurity was much wider than that of skill discretion and decision authority.*

***(page 14, line 308-312)***

*The slopes of the regression lines for migrants who arrived ≤5 years previously were close to zero; however, their 95% CIs largely overlapped with that of other groups. In the job insecurity―mental health relationships, the regression lines for all groups, including Australian-born workers and all migrant subgroups based on COB, dominant language of COB and years since arrival in Australia, were almost overlapping (see* ***Fig 4****).*

***Discussion (page 16, line 339-345)***

*Skill discretion and decision authority were positively associated with MHI-5 score for both Australian-born workers and most subgroups of migrants, but there were non-statistically significant suggestions that regression lines were fairly flat for the most recently arrived migrants. Job insecurity was negatively associated with the MHI-5 score for both Australian-born workers and all subgroups of migrant workers. Furthermore, job insecurity was a stronger determinant of mental health than skill discretion and decision authority in all workers, with both a larger magnitude of effect on MHI-5 score and a wider range of change in predicted MHI-5 score.*

**R2-17. The value of the constant is shown in Table 2, but not mentioned in the Results or Discussion sections, as far as I can see. Do the differences in the constant value indicate important differences between the subgroups?**

**Response:**

The regression constant represents the predicted MHI-5 score when the measured psychosocial exposure=0. However, because our focus in this study is the relationship between psychosocial job characteristics and mental health, we focused more on beta coefficients.

We have made use of the constant values in one small way in the Results section. The predicted MHI-5 score in the Results section was calculated by: the constant value + beta coefficient x psychosocial job characteristic score from minimum to maximum in the unadjusted model (e.g. for skill discretion: 71.01+0.40x2=71.81, 71.01+0.40x14=76.61).

***Results (Page 13, line 284-288)***

*Moreover, combined with the constant values (since gender, age and educational attainment were not confounders for psychosocial job characteristic―mental health relationships, here, we used the unadjusted model to make the analysis easy), the difference in predicted MHI-5 score between the minimum and maximum job characteristic scale values were 71.8—76.6 for skill discretion, 71.1—78.5 for decision authority, and 80.8—62.1 for job insecurity.*

***Discussion (Page 16, line 343-345)***

*Furthermore, job insecurity was a stronger determinant of mental health than skill discretion and decision authority in all workers, with both a larger magnitude of effect on MHI-5 score and a wider range of change in predicted MHI-5 score.*

**R2-18. I would suggest writing what kind of analyses the results of the table are based on.**

**Response:**

Thank you for this comment. We have revised the table title and added “Results of univariable and multivariable linear regressions” in the footnote of Table 2. Furthermore, according to your comment, we have revised figure legends to include the analysis methods as well. Please see our response to your major comment (R2-1).

***The corresponding revision to the text is (Page 13-14, line 292-295):***

***Table 2. Psychosocial job characteristics and mental health: Unadjusted and adjusted linear regression results (n=8969).***

| *Psychosocial job characteristics* | *Unadjusted* | | *Adjusted for gender* | | *Adjusted for age* | | *Adjusted for education* | | *Adjusted^﹫^* | |
| --- | --- | --- | --- | --- | --- | --- | --- | --- | --- | --- |
|  | ***Cons*** | ***Coef. (95%CI)*** | ***Cons*** | ***Coef. (95%CI)*** | ***Cons*** | ***Coef. (95%CI)*** | ***Cons*** | ***Coef. (95%CI)*** | ***Cons*** | ***Coef. (95%CI)*** |
| *Skill discretion* | *71.01* | *0.40 (0.28, 0.52) ^***^* | *72.25* | *0.39 (0.27, 0.51) ^***^* | *69.78* | *0.40 (0.27, 0.52) ^***^* | *70.93* | *0.41 (0.28, 0.53) ^***^* | *71.02* | *0.40 (0.28, 0.53) ^***^* |
| *Decision authority* | *69.91* | *0.41 (0.33, 0.48) ^***^* | *71.20* | *0.38 (0.31, 0.45) ^***^* | *69.42* | *0.38 (0.31, 0.45) ^***^* | *69.88* | *0.41 (0.33, 0.48) ^***^* | *70.71* | *0.36 (0.29, 0.44) ^***^* |
| *Job insecurity* | *83.92* | *-1.04 (-1.13, -0.96) ^***^* | *85.57* | *-1.07 (-1.15, -0.98) ^***^* | *82.36* | *-1.05 (-1.13, -0.97) ^***^* | *83.54* | *-1.05 (-1.13, -0.97) ^***^* | *83.97* | *-1.08 (-1.16, -0.99) ^***^* |

*Results of univariable and multivariable linear regressions; Mental health was measured by MHI-5 score; Cons was the constant value; Coef. was the beta coefficient; *** p<0.001; ﹫Adjusted model adjusted for gender, age and educational attainment simultaneously*

**R2-19. Based on the LR test results, the authors state that there was no statistical evidence confirming migrant status acting as effect modifiers of the job stressor exposure—mental health relationships. This is also the conclusion of the manuscript. However, the stratified analyses, presented in the figures, show that migrants who arrived ≤5 years previously differed from other groups. Also migrants born in a Non-English speaking country seem to differ somewhat from other groups. The stratified analyses, thus, indicate that there is effect modification. To me, all three job stressors seem to have less effect on mental health among migrants who arrived ≤5 years previously, compared to other groups. They also seem to be less vulnerable to high job insecurity. The lines of the Australian-born group generally seem to lie somewhat higher than the lines of other groups for the two job control subscales. Could this indicate an interesting result?**

**Response:**

Thank you for the comments. We have addressed the first part of the comments in our response to major comment R2-1 above. In response to the final point:

The regression lines of Australian-born workers were higher than that of migrant workers in skill discretion/decision authority―mental health associations, which indicate when the skill discretion or decision authority was at the same level, Australian-born workers may have a higher MHI-5 score than migrant workers. However, LR test results were non-significant in our study, which indicate the differences in these regression lines may have arisen by chance. In **Figs 2** and **3**, it showed that the 95% CIs of the regression lines were overlapping.

**R2-20. I am not a statistician, and will not speculate why the LR test did not indicate effect modification. However, I will just mention that results of effect modification analyses will depend on the measures used, therefore, Rothman prefer to use the term "effect MEASURE modification" (e.g. in the book Modern Epidemiology), indicating that you e.g. may find effect modification on an additive scale, but not on a relative scale, or vice versa. Is it correct to test effect modification in linear analysis by adding a multiplicative interaction term? Or should this preferably be tested in other ways?**

**Response:**

Thank you for your comments. For the LR test comment, we have addressed it in our response to major comment R2-1.

Yes, we agree with you it should be ‘effect-measure modification’. Our study is indeed a salient demonstration of the need for this term―because we measured the single construct of migrant status in three distinct ways. We have changed ‘effect modification’ to ‘effect-measure modification’ throughout the manuscript.

For the additivity/multiplicativity question: testing interaction with a product term in linear regression (as we have done) tests departure from additivity, however, in logistic regression it refers to interaction as departure from multiplicativity [26].

**Discussion**

**R2-21. The first sentence (lines 294-296) is not according to the results, as far as I can see, as mentioned above.**

**Response:**

We have addressed this comment in our response to major comment R2-1. This sentence is the conclusion of what we draw from our results.

**R2-22. The authors refer to a study by Hoppe indicating that migrants from some countries may experience that working conditions in the new country is much better (lines 327-328). The opposite may also be true, if the migrants' education is not approved in the new country, and they have to accept manual work with lower job control and higher job insecurity.**

**Response:**

Yes, we agree with you that migrants may be exposed to higher levels of adverse psychosocial job characteristics if their qualifications are not accepted in the host country and they have to do jobs with poor working conditions. But here we cited Hoppe to explain a possible reason why migrant workers are not more vulnerable than native-born workers even though they experienced higher levels of psychosocial exposures―comparing with their former working conditions, the working conditions now are better even it is worse than that of native-born workers―rather than why they experienced higher levels of exposures.

**R2-23. Page 16 lines 359-361: The authors state: "…apart from reducing occupational health inequalities of migrant workers, reducing job stressor exposures should improve mental health of Australia-born workers as well." This could easily result in occupational health inequalities being maintained.**

**Response:**

Yes, we agree that reducing adverse psychosocial exposures equally for all workers would preserve occupational mental health inequalities between migrant and native-born workers; however, the job characteristic-associated mental health burden would be reduced. Moreover, the recommendations we highlighted in our study were to reduce adverse psychosocial job characteristic exposures for migrants in particular by improving migrants’ English skill, which would be specifically helpful to reduce adverse psychosocial job characteristic exposures among migrant workers and, thus, reduce occupational mental health inequalities between migrant and native-born workers.

**R2-24. Lines 366-368: Maybe add that poor working conditions may lead to poor health, and therefore healthy worker survivor effect (there seemed to be a missing link).**

**Response:**

Thank you for your comment. We have revised this limitation.

***The corresponding revision to the text is (Page 19, line 409-411):***

*Second, our results may have been biased toward the null due to the healthy worker effect―some workers may have dropped out of the workforce due to poor working conditions thus underestimating exposure-outcome associations.*

**R2-25. Page 17, lines 373-375: Common method bias could also be a limitation of a study based on subjective/self-reported job stressors and mental health.**

**Response:**

Thank you for your comment. We have revised this limitation.

***The corresponding revision to the text is (Page 19, line 417-420):***

*Fourth, one limitation of our study is the reliance on self-report measures, both for psychosocial job characteristics and mental health, thus common method bias could potentially bias the relationship between psychosocial job characteristics and mental health.*

**References**

**R2-26. When referring to more than one authors in the text, the authors include two of the names, instead of using one name + et al. Is this according to the PLOS? For some references in the reference list, the journal titles are spelled out, while for others, the usual abbreviated forms are used. These should also be according to the style of the journal.**

**Response:**

Thank you for your comments. Yes, we have used the Endnote style of PloS One to insert the references in our previous manuscript. Now, we have revised the reference list.

**Language**

**R2-27. The language is generally good, but some sentences starting with "there is/were…" seem a bit incomplete (e.g. lines 101, 120, 122 and 125). A couple of places there is also an S missing in 3rd person present tense (lines 105 and 143). There are also a few other things. Line 365: Should "casual" be "causal"?**

**Response:**

Thank you for your comments. We have edited and proofread the manuscript carefully.

**References**

1.	Landsbergis PA, Grzywacz JG, LaMontagne AD. Work organization, job insecurity, and occupational health disparities. Am J Ind Med. 2014;57(5):495-515. doi: 10.1002/ajim.22126. PubMed PMID: 23074099.

2.	Centres for Disease Control and Prevention. Occupational health equity 2019 [updated 18 December 2019; cited 2020 18 February]. Available from: <https://www.cdc.gov/niosh/programs/ohe/default.html>.

3.	LaMontagne AD. Commentary: Precarious employment: Adding a health inequalities perspective. J Public Health Policy. 2010;31(3):312-7. doi: 10.1057/jphp.2010.25. PubMed PMID: 20805803.

4.	Abubakar I, Aldridge RW, Devakumar D, Orcutt M, Burns R, Barreto ML, et al. The UCL–Lancet Commission on Migration and Health: The health of a world on the move. Lancet. 2018;392(10164):2606-54. doi: 10.1016/S0140-6736(18)32114-7. PubMed PMID: 30528486.

5.	Quandt SA, Arcury-Quandt AE, Lawlor EJ, Carrillo L, Marín AJ, Grzywacz JG, et al. 3-D jobs and health disparities: The health implications of Latino chicken catchers' working conditions. Am J Ind Med. 2013;56(2):206-15. doi: 10.1002/ajim.22072. PubMed PMID: 22618638.

6.	Thamrin Y. A literature review of migrant workers’ health and safety. Jurnal Kesehatan Masyarakat Maritim. 2018;2(1):245-94.

7.	Hargreaves S, Rustage K, Nellums LB, McAlpine A, Pocock N, Devakumar D, et al. Occupational health outcomes among international migrant workers: A systematic review and meta-analysis. The Lancet Global Health. 2019;7(7):e872-e82. doi: 10.1016/S2214-109X(19)30204-9. PubMed PMID: 31122905.

8.	Sterud T, Tynes T, Mehlum IS, Veiersted K, Bergbom B, Airila A, et al. A systematic review of working conditions and occupational health among immigrants in Europe and Canada. BMC Public Health. 2018;18(1):770. doi: 10.1186/s12889-018-5703-3. PubMed PMID: 29925349.

9.	WHO. Mental health in the workplace 2019 [updated May 2019; cited 2020 1 February ]. Available from: <https://www.who.int/mental_health/in_the_workplace/en/>.

10.	OECD. Fit mind, fit job: From evidence to practice in mental health and work Paris: OECD Publishing; 2015.

11.	Stansfeld S, Candy B. Psychosocial work environment and mental health—a meta-analytic review. Scand J Work Environ Health. 2006;32(6):443-62.

12.	Llosa JA, Menéndez-Espina S, Agulló-Tomás E, Rodríguez-Suárez J. Job insecurity and mental health: A meta-analytical review of the consequences of precarious work in clinical disorders. Anales de Psicología. 2018;34(2):211-23. doi: 10.6018/analesps.34.2.281651.

13.	Saijo Y, Yoshioka E, Kawanishi Y, Nakagi Y, Itoh T, Sugioka Y, et al. Effects of work burden, job strain and support on depressive symptoms and burnout among Japanese physicians. Int J Occup Med Environ Health. 2014;27(6):980-92. doi: 10.2478/s13382-014-0324-2. PubMed PMID: edselc.2-52.0-84916912162.

14.	Theorell T, Hammarström A, Aronsson G, Träskman Bendz L, Grape T, Hogstedt C, et al. A systematic review including meta-analysis of work environment and depressive symptoms. BMC Public Health. 2015;15:738. doi: 10.1186/s12889-015-1954-4. PubMed PMID: 26232123.

15.	Milner A, Witt K, LaMontagne AD, Niedhammer I. Psychosocial job stressors and suicidality: A meta-analysis and systematic review. Occup Environ Med. 2018;75(4):245-53. doi: 10.1136/oemed-2017-104531. PubMed PMID: 28851757.

16.	Milner A, LaMontagne AD, Spittal MJ, Pirkis J, Currier D. Job stressors and employment precarity as risks for thoughts about suicide: An Australian study using the Ten to Men Cohort. Ann Work Expo Health. 2018;62(5):583-90. doi: 10.1093/annweh/wxy024. PubMed PMID: 29635407.

17.	Milner A, Smith P, LaMontagne AD. Working hours and mental health in Australia: Evidence from an Australian population-based cohort, 2001–2012. Occup Environ Med. 2015;72(8):573-9. doi: 10.1136/oemed-2014-102791. PubMed PMID: 26101295.

18.	LaMontagne AD, Milner A, Krnjacki L, Kavanagh AM, Blakely TA, Bentley R. Employment arrangements and mental health in a cohort of working Australians: Are transitions from permanent to temporary employment associated with changes in mental health? Am J Epidemiol. 2014;179(12):1467-76. doi: 10.1093/aje/kwu093. PubMed PMID: 103963370.

19.	LaMontagne AD, Krnjacki L, Kavanagh AM, Bentley R. Psychosocial working conditions in a representative sample of working Australians 2001-2008: An analysis of changes in inequalities over time. Occup Environ Med. 2013;70(9):639-47. doi: 10.1136/oemed-2012-101171. PubMed PMID: 23723298.

20.	Australian Bureau of Statistics. ANZSCO - Australian and New Zealand Standard Classification of Occupations, First Edition. Canberra, Australia: ABS, 2006.

21.	Watson N, Wooden M. The Household, Income and Labour Dynamics in Australia (HILDA) survey: Wave 1 survey methodology. Melbourne: Melbourne Institute of Applied Economic and Social Research, University of Melbourne, 2002.

22.	Summerfield M, Freidin S, Hahn M, La N, Li N, Macalalad N, et al. HILDA user manual-Release 15. Melbourne: Melbourne Institute of Applied Economic and Social ResearchResearch, University of Melbourne, 2016.

23.	Hayes AF. Introduction to mediation, moderation, and conditional process analysis: A regression-based approach. Second ed. New York: Guilford publications; 2018. 223-66 p.

24.	Liu X, Bowe SJ, Milner A, Li L, Too LS, Lamontagne AD. Differential exposure to job stressors: A comparative analysis between migrant and Australia-born workers. Ann Work Expo Health. 2019;63(9):975–89. doi: 10.1093/annweh/wxz073. PubMed PMID: 31621876.

25.	Liu X, Bowe SJ, Milner A, Li L, Too LS, LaMontagne AD. Job insecurity: A comparative analysis between migrant and native workers in Australia. Int J Environ Res Public Health. 2019;16(21):4159. doi: 10.3390/ijerph16214159. PubMed PMID: 31661926.

26.	Knol MJ, van der Tweel I, Grobbee DE, Numans ME, Geerlings MI. Estimating interaction on an additive scale between continuous determinants in a logistic regression model. Int J Epidemiol. 2007;36(5):1111-8. doi: 10.1093/ije/dym157.
