# Supplementary material for: Psychosocial job characteristics and mental health: Do associations differ by migrant status in an Australian working population sample?
Source: PLoS One. 2020 Nov 30;15(11):e0242906. doi: 10.1371/journal.pone.0242906 (PMC7703972; doi:10.1371/journal.pone.0242906)
Supplement: Attachment — Submitted filename: Response to Reviewers.docx [file pone.0242906.s009.docx]

**Academic Editor**

**AE-1. Reviewer 2 asks for more clarification with respect to the data analysis in general and the method to test migrant as an effect modifier in particular. I suggest to present in additional analyses the robustness and non-significance of the interaction effect. The additional analyses do not have to be part of the manuscript; a presentation in the response letter should suffice.**

**Response:**

Thank you for your suggestion. We have added a supplemental table to report slopes of regression lines for the figures in this revised manuscript. We also reported the differences in slopes between migrant subgroups and Australian-born workers to Reviewer 2. Please see the response to R2-1. If the reviewer remains unsatisfied with our analytic approach to effect-measure modification, we would suggest a Statistical Reviewer. We stand by our analysis as appropriate, and our interpretation as correct. It is indeed an unusual situation for the authors to be arguing for a more conservative interpretation than a Reviewer(!).

***The corresponding revisions to the text are:***

1) *Results section (page 15, line 318-319):*

*……The slopes of the regression lines in* ***Figs 2-4*** *were reported in the supporting information* ***S3 Table.***

2) *Supporting information* ***S3 Table****:*

***S3 Table.*** *The slopes of regression lines for relationships between psychosocial job characteristics and mental health: stratified by migrant status and controlled for gender, age and educational attainment*

| ***Psychosocial job characteristics*** | ***Migrant status*** | ***Predicted mental health*** | | | |
| --- | --- | --- | --- | --- | --- |
|  |  | ***Coefficient*** | ***SE*** | ***P value*** | ***95% CI*** |
| ***Skill discretion*** | *Australian-born* | *0.38* | *0.07* | *0.00* | *0.24; 0.51* |
|  | *Overseas-born* | *0.48* | *0.14* | *0.001* | *0.20; 0.75* |
|  | *Main-ESC-born^†^* | *0.37* | *0.21* | *0.08* | *-0.04; 0.78* |
|  | *Non-ESC-born^†^* | *0.55* | *0.19* | *0.003* | *0.19; 0.92* |
|  | *Arrived ≤5 years* | *0.10* | *0.44* | *0.827* | *-0.76; 0.95* |
|  | *Arrived 6-10 years* | *0.58* | *0.38* | *0.125* | *-0.16; 1.34* |
|  | *Arrived ≥11 years* | *0.51* | *0.16* | *0.001* | *0.20; 0.83* |
| ***Decision authority*** | *Australian-born* | *0.36* | *0.04* | *0.00* | *0.28; 0.44* |
|  | *Overseas-born* | *0.37* | *0.08* | *0.00* | *0.21; 0.53* |
|  | *Main-ESC-born^†^* | *0.27* | *0.12* | *0.03* | *0.03; 0.50* |
|  | *Non-ESC-born^†^* | *0.47* | *0.12* | *0.00* | *0.24; 0.70* |
|  | *Arrived ≤5 years* | *-0.04* | *0.35* | *0.90* | *-0.73; 0.65* |
|  | *Arrived 6-10 years* | *0.58* | *0.25* | *0.022* | *0.08; 1.08* |
|  | *Arrived ≥11 years* | *0.37* | *0.09* | *0.00* | *0.19; 0.55* |
| ***Job insecurity*** | *Australian-born* | *-1.08* | *0.05* | *0.00* | *-1.18; -1.00* |
|  | *Overseas-born* | *-1.03* | *0.09* | *0.00* | *-1.21; -0.85* |
|  | *Main-ESC-born^†^* | *-0.99* | *0.14* | *0.00* | *-1.26; -0.99* |
|  | *Non-ESC-born^†^* | *-1.07* | *0.12* | *0.00* | *-1.31; -0.83* |
|  | *Arrived ≤5 years* | *-0.79* | *0.33* | *0.02* | *-1.44; -0.14* |
|  | *Arrived 6-10 years* | *-1.27* | *0.27* | *0.00* | *-1.80; -0.74* |
|  | *Arrived ≥11 years* | *-1.03* | *0.10* | *0.00* | *-1.24; -0.83* |

*† Main-ESC-born: Born in Main-English-speaking country, Non-ESC-born: Born in Non-English-speaking country; Mental health was measured by MHI-5 score*

**AE-2. It was mentioned in the response letter that you adopted the job demand-control (JDC) model as theoretical framework for the study. I neither saw this clearly implemented in the manuscript nor do I believe that the JDC model would be enough because migrant status cannot be conceptualised as effect modifier by means of this model (alone).**

**Response:**

Thank you for your comments. We adopted the Job Demand-Control (JDC) model to define the psychosocial job characteristics; however, we conceptualised migrant status as a potential determinant of adverse occupational exposures based on the framework developed by Landsbergis *et al.* (1) (*Page 3, line 56-59; Supporting information* ***S1 Fig***), through which migrant status may act as an effect modifier of the relationship between psychosocial job characteristics and mental health. We have now explained this further in the Methods section.

***The corresponding revision to the text is (page 7, line154-156):***

*Psychosocial job characteristics were the exposure variables, which were assessed based on the Job Demand-Control model [2] and skill discretion, decision authority and job insecurity were measured in this study.*

**Reviewer 2**

**R2-1. I have reviewed this manuscript previously. The authors have responded adequately to most of my comments, and they have also addressed my major concern. However, I am still not convinced that the conclusion is correct.**

**Response:**

We do agree with the reviewer that the figures (non-statistical evidence) *suggested* there may be effect-measure modification; however, the statistical evidence and the largely overlapping 95% CI of the slopes of regression lines did not support that finding. We appreciate the reviewer’s advocacy for this finding, but we do not want to overstate. We do, however, believe the non-statistical evidence (differences in the slopes) suggests that our research questions were not fully answered at this stage, and that future studies with larger sample sizes and higher statistical power would serve to answer the question more definitively. This was the reason why we kept these figures in this manuscript even though the statistical evidence suggested no effect modification. Furthermore, it is important to note that testing effect modification is different from stratification.

To further substantiate our finding that we do *not* have statistical confirmation of effect-measure modification, the slopes with 95% CI of regression lines of our figures are presented in supporting information **S3 Table**. Here, we also reported the differences in the slopes between migrant subgroups and Australian-born workers (see **Table 1**). It showed that the 95% CIs of the slopes for the migrants ≤5 years since arrival were so wide that they almost completely overlapped with those of the Australian-born workers. This indicated that these slopes are *not* statistically different from each other. This is likely due to the relatively small sample size of migrants ≤5 years since arrival (n=154) compared to the sample size of Australian-born workers (n=7238). None of the differences in the slopes between migrant subgroups and Australian-born workers were statistically different from zero, which has been supported by the LR test results.

**Table 1.** Relationships between psychosocial job characteristics and mental health stratified by migrant status and the differences in the slopes between each migrant subgroup and Australian-born workers (controlled for gender, age and educational attainment)

| **Psychosocial job characteristics** |  | **Migrant status** | **Predicted mental health** | | | |
| --- | --- | --- | --- | --- | --- | --- |
|  |  |  | **Coefficient** | **SE** | **P value** | **95% CI** |
| **Skill discretion** | The slope of each subgroup | Australian-born | 0.38 | 0.07 | 0.00 | 0.24; 0.51 |
|  |  | Overseas-born | 0.48 | 0.14 | 0.001 | 0.20; 0.75 |
|  |  | Main-ESC-born^†^ | 0.37 | 0.21 | 0.08 | -0.04; 0.78 |
|  |  | Non-ESC-born^†^ | 0.55 | 0.19 | 0.003 | 0.19; 0.92 |
|  |  | Arrived ≤5 years | 0.10 | 0.44 | 0.827 | -0.76; 0.95 |
|  |  | Arrived 6-10 years | 0.58 | 0.38 | 0.125 | -0.16; 1.34 |
|  |  | Arrived ≥11 years | 0.51 | 0.16 | 0.001 | 0.20; 0.83 |
|  | Differences in the slopes | Overseas-born - Australian-born | 0.10 | 0.16 | 0.00 | -0.20; 0.41 |
|  |  | Main-ESC-born^†^ - Australian-born | -0.01 | 0.22 | 0.98 | -0.44; 0.43 |
|  |  | Non-ESC-born^†^ - Australian-born | 0.18 | 0.20 | 0.38 | -0.21; 0.57 |
|  |  | Arrived ≤5 years - Australian-born | -0.28 | 0.44 | 0.52 | -1.14; 0.58 |
|  |  | Arrived 6-10 years - Australian-born | 0.21 | 0.39 | 0.60 | -0.56; 0.97 |
|  |  | Arrived ≥11 years - Australian-born | 0.13 | 0.17 | 0.45 | -0.21; 0.47 |
| **Decision authority** | The slope of each subgroup | Australian-born | 0.36 | 0.04 | 0.00 | 0.28; 0.44 |
|  |  | Overseas-born | 0.37 | 0.08 | 0.00 | 0.21; 0.53 |
|  |  | Main-ESC-born^†^ | 0.27 | 0.12 | 0.03 | 0.03; 0.50 |
|  |  | Non-ESC-born^†^ | 0.47 | 0.12 | 0.00 | 0.24; 0.70 |
|  |  | Arrived ≤5 years | -0.04 | 0.35 | 0.90 | -0.73; 0.65 |
|  |  | Arrived 6-10 years | 0.58 | 0.25 | 0.022 | 0.08; 1.08 |
|  |  | Arrived ≥11 years | 0.37 | 0.09 | 0.00 | 0.19; 0.55 |
|  | Differences in the slopes | Overseas-born - Australian-born | 0.01 | 0.09 | 0.90 | -0.17; 0.19 |
|  |  | Main-ESC-born^†^ - Australian-born | -0.09 | 0.13 | 0.45 | -0.34; 0.15 |
|  |  | Non-ESC-born^†^ - Australian-born | 0.11 | 0.12 | 0.37 | -0.13; 0.36 |
|  |  | Arrived ≤5 years - Australian-born | -0.40 | 0.35 | 0.26 | -1.09; 0.29 |
|  |  | Arrived 6-10 years - Australian-born | 0.22 | 0.26 | 0.39 | -0.28; 0.73 |
|  |  | Arrived ≥11 years - Australian-born | 0.01 | 0.10 | 0.91 | -0.18; 0.21 |
| **Job insecurity** | The slope of each subgroup | Australian-born | -1.08 | 0.05 | 0.00 | -1.18; -1.00 |
|  |  | Overseas-born | -1.03 | 0.09 | 0.00 | -1.21; -0.85 |
|  |  | Main-ESC-born^†^ | -0.99 | 0.14 | 0.00 | -1.26; -0.99 |
|  |  | Non-ESC-born^†^ | -1.07 | 0.12 | 0.00 | -1.31; -0.83 |
|  |  | Arrived ≤5 years | -0.79 | 0.33 | 0.02 | -1.44; -0.14 |
|  |  | Arrived 6-10 years | -1.27 | 0.27 | 0.00 | -1.80; -0.74 |
|  |  | Arrived ≥11 years | -1.03 | 0.10 | 0.00 | -1.24; -0.83 |
|  | Differences in the slopes | Overseas-born - Australian-born | 0.05 | 0.10 | 0.64 | -0.15; 0.25 |
|  |  | Main-ESC-born^†^ - Australian-born | 0.10 | 0.15 | 0.51 | -0.19; 0.39 |
|  |  | Non-ESC-born^†^ - Australian-born | 0.01 | 0.13 | 0.93 | -0.25; 0.27 |
|  |  | Arrived ≤5 years - Australian-born | 0.29 | 0.34 | 0.387 | -0.37; 0.95 |
|  |  | Arrived 6-10 years - Australian-born | -0.19 | 0.27 | 0.49 | -0.72; 0.35 |
|  |  | Arrived ≥11 years - Australian-born | 0.05 | 0.11 | 0.66 | -0.17; 0.27 |

† Main-ESC-born: Born in Main-English-speaking country, Non-ESC-born: Born in Non-English-speaking country; Mental health was measured by MHI-5 score

**R2-2. In fig. 2 (Skill discretion), to the right, the change for Australian-born workers is from a score of approx. 72 for low Skill discretion, to a score of approx. 76 for high Skill discretion, i.e. a score change of approx. 4. For migrants with ≤ 5 years since arrival, the score change is only about 1 (73 to 74), while for migrants > 5 years since arrival, the difference is larger than for Australian-born workers. I can hardly believe that the difference between Australian-born (score change 4) and migrants with ≤ 5 years since arrival (score change 1) is not statistically significant, even though the latter group is small. The figure shows very different curves for the two groups.**

**Response:**

Statistical testing effect modification of migrant status is to assess whether the differences in the associations between psychosocial job characteristics and mental health (slopes of regression lines)―rather than the differences in the changes in predicted MHI-5 scores with psychosocial job characteristics changing from the minimum to the maximum―between migrant subgroups and Australian-born workers are significantly different from zero. As we responded in comment R2-1, none of the differences in slopes were significantly different from zero.

We showed the three graphs side by side in our paper to facilitate comparison of the results for the three different measures of migrant status used. The combining of the three figures compressed the x-axis, and the y-axis had a scale from 60 to 80―which makes the differences in slopes clearly, but possibly makes the changes in predicted MHI-5 scores look more dramatic than they would with wider scaling. In **Figure 1** below (Figure 1 in Response document), we showed only one graph and the y-axis extended to range from 40 to100. It still showed that the slopes of regression line for migrants ≤5 years since arrival was almost flat and all 95% CIs were overlapping. Now, we have revised the Results section in our manuscript by deleting the changes in the predicted MHI-5 scores to avoid possible confusion.

**Figure 1.** Relationships between skill discretion and mental health stratified by migrant status based on years since arrival

**R2-3. The authors write (lines 329-330): Since the LR tests were not significant, the relationships between psychosocial job characteristics and mental health were not stratified by migrant status. Is the performed LR test the best way of evaluating effect measure modification? Do stratified analysis confirm the results of the LR test?**

**Response:**

There are a number of ways to determine whether an interaction (effect modifier) is present, such as the LR test, global test (F statistic test), or comparing adjusted R squared value, BIC and AIC values between models with and without interaction term. We used the LR test in our study. We also conducted the other two tests; both of which indicated there was no significant effect-measure modification.

For example, the LR test result for whether years since arrival moderated the relationship between skill discretion and mental health was not significant (chi2(3)=1.32, p=0.72). The global test also reported a non-significant result (F(3,8953)=0.44, p=0.72). Finally, the adjusted R squared value was larger (0.0168 vs. 0.0166), and AIC and BIC were smaller (AIC: 75075.48 vs. 75080.16 and BIC: 75167.80 vs. 75193.78) for the model without interaction term. All three of the tests indicated that the model without interaction term was a better model.

The LR test provides evidence whether the stratification was justified or not, and stratification is not justified based on the results of the LR test in our study. We have revised the Results section according to the reviewer’s comments. We understood this revision included some discussion of the content, but we prefer to keep it in the Results section in the hope of clarifying why we did not stratify.

***The corresponding revision to the text is (page 15-16, line 336-340):***

*Because none of the LR test results were statistically significant, there was no justification to stratify the psychosocial job characteristic―mental health relationships by migrant status. This suggests that the differences in the magnitude of the relationships between psychosocial job characteristic and mental health―which were also shown as the differences in the slopes of regression lines in* ***Figs 2-4****―between migrant subgroups and Australian-born workers were not statistically significantly different from zero.*

**R2-4. In this LR test, the interaction term consists of a psychosocial job characteristic variable, which is continuous, and a migrant status variable, which is categorical with 4 categories. Is the migrant status variable treated correctly in the interaction analysis? Often the variables in an interaction term are either continuous or dichotomous (as mentioned in ref. 26 in the response to the reviewers). How does the analysis treat the migrant status variable? As a categorical variable (with no intrinsic ordering to the categories)? As an ordinal variable (with clear ordering of the categories)? As a numerical variable (with equal intervals between the values of the variable)? Or as a continuous variable? Are all these alternatives possible in an LR analysis, e.g. categorical variables? Are all assumptions met for this analysis?**

**Response:**

The migrant status in our study is a categorical variable without intrinsic ordering. The regression-based analysis for effect modification has no restriction on the nature of exposure variables and potential effect modifiers [3], and categorical variable can have more than two categories in testing effect modification [4].

**R2-5. What do the partly overlapping confidence intervals indicate? If the constant is very similar but the slope very different, they would probably partly overlap, but does it mean that they are not significantly different?**

**Response:**

The 95% confidence interval shows the upper and lower limits of the slope of regression lines of each subgroup based on migrant status. The large overlap indicates that these slopes are not statistically significantly different from each other. When there is effect modification, the lines may cross―which means the 95% CIs could overlap. The slopes would still be statistically significantly different from each other if the overlapping areas are not large.

**R2-6. If I understand correctly, Australian-born workers are the reference group that different migrant groups are compared to. However, this is not always clear.**

**Response:**

Yes, Australian-born workers were always the reference group when compared with migrant workers in this study and thus, we did not state this specifically in each instance. However, we hypothesised that Non-ESC-born and newly arrived migrants would be the most vulnerable migrant subgroups, therefore, where we noticed clear differences between migrant subgroups, we would report that as well. We have now revised our manuscript to add the reference groups wherever possible.

***The corresponding revisions of the text are:***

*Introduction section (page 4, line 91-93)*

*However, results to the question of whether migrant workers are more sensitive to psychosocial job characteristics in terms of mental health compared to native-born workers are inconsistent.*

*Introduction section (page 5, line 116-117)*

*……, whether migrants are differentially affected by psychosocial job characteristic-associated impacts on mental health compared to Australian-born workers is unclear,……*

*Results section (page 14, line 311-315)*

*……, the regression lines for migrants who arrived ≤5 years previously appeared to differ from the lines for Australian-born workers and other migrant subgroups as well. The slopes of the regression lines for migrants who arrived ≤5 years previously were close to zero; however, their 95% CIs largely overlapped with that of Australian-born workers and other migrant subgroups.*

*Discussion section*

*(page 16, line 346-349)*

*However, there was a suggestion that psychosocial job characteristic―mental health associations for migrants who had arrived in Australia ≤5 years previously differed from the associations for Australian-born workers and migrants who had been living in Australia for longer,……*

*(page 17, line 376-377)*

*Various explanations as to why migrants would not have a higher vulnerability to psychosocial job characteristics than native-born workers are plausible.*

**R2-7. TITLE: The title of the manuscript has been changed, however, I now find it difficult to read. The term "psychosocial job characteristic-associated impacts" describes the outcome, but is very long and complicated.**

**Response:**

Thank you for this suggestion. We have now revised the Title.

***The corresponding revision of the text is:***

*Psychosocial job characteristics and mental health: do associations differ by migrant status in an Australian working population sample?*

**R2-8. INTRODUCTION: Lines 52-57 (and elsewhere): The term "occupational health inequalities" is used to describe one or specific groups in this paragraph, without referring to which groups they are compared to. Inequalities/differences - compared to which groups?**

**Response:**

In some situations, such as what we wrote in *line 54-59*, it was not difficult to understand the reference groups but difficult to mention all of them. Therefore, we did not list the reference groups one by one. Furthermore, with Australian-born workers always being the reference group in this study, we did not repeat it each time. We have incorporated the reviewer’s comment by adding the reference groups wherever possible in this revised manuscript.

***The corresponding revisions of the text are:***

*Discussion section*

*(page 19, line 412-413)*

*……, reducing job insecurity would seem to be the most appropriate target for reducing the risk of OHIs between migrant workers and Australian-born workers.*

*(page 19, line 415-416)*

*……, continuing language support may be helpful to reduce OHIs experienced by migrants compared to Australian-born workers.*

**R2-9. Line 75: Some of the psychosocial job characteristics are referred to as high or low, but this could also apply to job strain and job insecurity.**

**Response:**

Thank you for this comment. We have revised this sentence in the Introduction section.

***The corresponding revision to the text is (Page 4, line 75-78):***

*Adverse psychosocial job characteristics―including high job demands, low job control, high job strain, high job insecurity, effort-reward imbalance and lack of social support at work―have been shown to be associated with a wide range of adverse mental health outcomes……*

**R2-10. Lines 116-118: "We hypothesise that the psychosocial job characteristic―mental health relationships are stronger for migrants, especially … migrants recently arrived in Australia." Why do you hypothesise this? Isn't it contrary to studies you refer to?**

**Response:**

Following the occupational health inequality (OHI) framework of Landsbergis *et al.* (1), disadvantaged population groups, such as migrants, are more likely to experience OHIs due to higher exposure and/or higher vulnerability. Moreover, previous studies have shown that disadvantaged population groups, such as workers in the lowest socioeconomic positions [5], were more vulnerable to psychosocial job characteristic-associated mental health impacts.

However, findings of most previous studies showed that compared to native-born workers, migrant workers have similar or even weaker vulnerability to mental health impacts of exposure to psychosocial job characteristics. We were mindful of the shortcomings of the simplistic measures of migrant status used in most of the previously published literature. We considered that if years since arrival or language facility in the destination country were taken into account, such a measure of migrant status might show the hypothesised direction of relationship: 1) recent migrants could plausibly be less acculturated in the destination country, they would have had less time to develop social networks and supports to buffer the effects of stressors, and they might also be precarious in terms of housing and financial security; 2) migrants with low English skills would more vulnerable again because it might impede social integration and social connections, and non-Caucasian workers might also experience racism at work or in the general community.

Working from general principles drawing from Landsbergis *et al.* (1) combined with our more detailed characterisation (and measures) of migrant status, we anticipated/hyothesised that we would see stronger exposure-outcome associations among migrant workers compared to native-born workers. We now have revised the Introduction section of our manuscript to explain our hypothesis establishment clearer.

***The corresponding revisions to the text are:***

*(Page 4, line 79-83)*

*……**Adverse psychosocial job characteristics……have been shown to be associated with a wide range of adverse mental health outcomes……However, the vulnerability to psychosocial job characteristic-associated mental health impacts may be different between working population groups―those who are disadvantaged have been reported to be more vulnerable. For example, compared to workers with higher socioeconomic status, those with lower socioeconomic status showed stronger associations between effort-reward imbalance and depression, as well as job strain and depression [5].*

*(Page 6, line 123-127)*

*Based on that migrant workers are usually characterised as a disadvantaged population group and thus may be more vulnerable to psychosocial job characteristic-associated mental health impacts, we hypothesise that the psychosocial job characteristic―mental health relationships are stronger for migrants, especially migrants from Non-English-speaking countries, and migrants recently arrived in Australia.*

**R2-11. MATERIAL AND METHODS: Line 191: "8983 observations answered…" Can an observation answer?**

**Response:**

Thank you for this comment. We have revised this sentence.

***The corresponding revision to the text is (Page 9, line 202-203):***

*……8983 observations had completed data on all the items of the three psychosocial job characteristic scales,……*

**R2-12. Lines 204-206: I suggest moving this sentence ("moreover, a small sample…") to the end of the paragraph, so that the last sentence ("This left 86 respondents…", lines 206-208) comes closer to the sentence "Among the 95 who refused to answer, 9 were missing all items…" (line 202), i.e. the 86 referred to in the current last sentence.**

**Response:**

Thank you for this comment. We have revised this paragraph.

***The corresponding revision to the text is (Page 10, line 214-218):***

*……Among the 95 who refused to answer, 9 were missing all items and 86 were missing one or two items for the three psychosocial job characteristic scales. The number who were missing one or two items for the psychosocial job characteristic scales (n=86) was much smaller with respect to the number who were missing all items (n=1184+286+9=1479) and thus, limiting possibilities for value substitution (e.g., mean substitution of single missing items).*

**R2-13. RESULTS: Lines 256-264: I suggest reversing the comparisons (if Australian-born workers are the reference). E.g., instead of "Australian-born workers included a significantly lower proportion of males than Main-ESC-born workers", I suggest: "Main-ESC-born workers included a significantly higher proportion of males than Australian-born workers".**

**Response:**

Thank you for this comment. We have revised the comparisons.

***The corresponding revision to the text is (Page 12, line 266-273):***

*The differences in gender, age and educational attainment between migrant workers and Australian-born workers were apparent (all p-values <0.001). For example, Main-ESC-born workers included a significantly higher proportion of males than Australian-born workers (57.3% vs. 50.1%). Migrants who arrived in Australia between 6 to 10 years previously included a significantly higher proportion of workers with a postgraduate degree compared to Australian-born workers (30.2% vs. 11.8%). In contrast, overseas-born workers included a significantly lower proportion of workers between 15 to 24 years of age than Australian-born workers (6.3% vs. 20.5%).*

**R2-14. In order to better understand and interpret the results, I would recommend adding a table showing the distribution of exposures and outcome (e.g. mean and range), according to migrant categories.**

**Response:**

The research question in this study was to test whether migrant status modified the relationships between psychosocial job characteristics and mental health. The distributions of psychosocial job characteristics and mental health by migrant status were not related to the moderation test; thus, we believe this would be beyond the scope of our paper. Nevertheless, we have presented these results in a supplementary table (**S1 Table**).

***The corresponding revision to the text is (Page 13, line 278-279):***

*Distributions of psychosocial job characteristics and MHI-5 scores by migrant status were reported in supporting information* ***S1 Table****.*

***S1 Table****. Distributions of psychosocial job characteristics and MHI-5 scores by migrant status*

|  | ***Migrant status*** | ***Minimum*** | ***Maximum*** | ***Median*** | ***Mean (Standard error)*** |
| --- | --- | --- | --- | --- | --- |
| ***Skill discretion*** | *Australian-born* | *2* | *14* | *10* | *10.00 (2.76)* |
|  | *Overseas-born* | *2* | *14* | *10* | *9.95 (2.73)* |
|  | *Main-ESC-born^†^* | *2* | *14* | *10* | *10.14 (2.66)* |
|  | *Non-ESC-born^†^* | *2* | *14* | *10* | *9.79 (2.78)* |
|  | *Arrived ≤5 years* | *2* | *14* | *10* | *9.69 (2.95)* |
|  | *Arrived 6-10 years* | *2* | *14* | *10* | *10.00 (2.94)* |
|  | *Arrived ≥11 years* | *2* | *14* | *10* | *9.98 (2.67)* |
| ***Decision authority*** | *Australian-born* | *3* | *21* | *12* | *12.44 (4.66)* |
|  | *Overseas-born* | *3* | *21* | *13* | *13.06 (4.57)* |
|  | *Main-ESC-born^†^* | *3* | *21* | *14* | *13.26 (4.72)* |
|  | *Non-ESC-born^†^* | *3* | *21* | *13* | *12.89 (4.42)* |
|  | *Arrived ≤5 years* | *3* | *21* | *12* | *11.91 (3.64)* |
|  | *Arrived 6-10 years* | *3* | *21* | *13* | *12.50 (4.43)* |
|  | *Arrived ≥11 years* | *3* | *21* | *14* | *13.27 (4.65)* |
| ***Job insecurity*** | *Australian-born* | *3* | *21* | *8* | *8.47 (3.83)* |
|  | *Overseas-born* | *3* | *21* | *9* | *8.91 (4.02)* |
|  | *Main-ESC-born^†^* | *3* | *21* | *9* | *8.64 (3.89)* |
|  | *Non-ESC-born^†^* | *3* | *21* | *9* | *9.14 (4.13)* |
|  | *Arrived ≤5 years* | *3* | *21* | *10* | *10.00 (3.73)* |
|  | *Arrived 6-10 years* | *3* | *21* | *9* | *9.28 (4.05)* |
|  | *Arrived ≥11 years* | *3* | *21* | *9* | *8.74 (4.03)* |
| ***MHI-5 score*** | *Australian-born* | *0* | *100* | *80* | *75.12 (16.00)* |
|  | *Overseas-born* | *4* | *100* | *80* | *74.50 (16.24)* |
|  | *Main-ESC-born^†^* | *4* | *100* | *80* | *75.12 (16.16)* |
|  | *Non-ESC-born^†^* | *8* | *100* | *76* | *73.96 (16.30)* |
|  | *Arrived ≤5 years* | *24* | *100* | *76* | *72.86 (16.56)* |
|  | *Arrived 6-10 years* | *16* | *100* | *80* | *74.04 (16.04)* |
|  | *Arrived ≥11 years* | *4* | *100* | *80* | *74.74 (16.23)* |

*† Main-ESC-born: Born in Main-English-speaking country, Non-ESC-born: Born in Non-English-speaking country*

**R2-15. Lines 284-285: "since gender, age and educational attainment were not confounders for psychosocial job characteristic―mental health relationships". According to the model (S2 Fig), they were confounders, but they barely confounded the relationships, according to the results, i.e. they were only weak confounders.**

**Response:**

In our study, these demographic factors only resulted in minimal changes to the coefficients (as shown in **Table 2**), therefore, it can be argued they were not confounders in this study, but they were included as covariates anyways for completeness. Because we have deleted the results regarding the changes in predicted MHI-5 scores with psychosocial job characteristics changing from the minimum to the maximum in this revised manuscript, the sentence the reviewer mentioned in this comment has been deleted as well.

**R2-16. DISCUSSION: Lines 359-361: "… evidence that the most recently arrived migrants may not be sensitive to skill discretion and decision authority, suggesting associations for these two psychosocial job characteristics are stronger in Australian-born workers". You have not actually compared the two groups statistically, which I would recommend that you do.**

**Response:**

In our manuscript, we wrote ‘*We do, however, find suggestive, non-statistically significant evidence that the most recently arrived migrants may not be sensitive to skill discretion and decision authority, suggesting associations for these two psychosocial job characteristics are stronger in Australian-born workers (see* ***Figs 2 and 3****); however, these apparent differences may be due to the small sample size of the subgroup of migrants who arrived in Australia ≤5 years previously (n=154). Future study with a larger sample size would be required to resolve this question*’ (*page 17, line 369-375*). We have presented this as suggestive and explained the possible explanation and the need for further study.

Our rationale for this discussion has been presented in the response to comment R2-1. Please see our response to R2-1.

**R2-17. Lines 366-367: "Various explanations as to why migrants would not have a higher vulnerability to psychosocial job characteristics are plausible." You mention "healthy immigrant effect" and some other possible explanations. It is also worth mentioning that demographics of the most recently arrived migrants appear to be quite different, compared to the Australian-born workers: 55 % are 25-34 years of age (23 % of Australian-born), and 57 % are bachelor or postgraduates (29 % of Australian-born). Although you have adjusted for age and education level, these differences may also indicate other differences, which are not adjusted for. "Healthy immigrant effect" may partly cover this.**

**Response:**

Thank you for this comment. The demographic factors could act as both confounders and effect modifiers for the psychosocial job characteristic―mental health relationships. In this study, we have controlled them as confounders, which indicated that their effects on the relationships could be seen as being “removed”. Thus, we prefer not to discuss their effects specifically. However, as the reviewer said, they could be effect modifiers as well, but we did not test this in the current study due to the small sample size of some subgroups. Accordingly, we have added this as a limitation in the Discussion section.

***The corresponding revision to the text is (Page 20, line 437-441):***

*Some limitations should be considered in the interpretation of our findings……. Finally, our statistical power to assess effect-measure modification was limited by the relatively small sample sizes of subgroups, which not only resulted in the wide 95% CI of small subgroups but also prevented the three-way effect modification tests being conducted―in which the question of whether gender, age and educational attainment modified the moderation effect of migrant status on the relationships between psychosocial job characteristics and mental health would be tested.*

**R2-18. Lines 390-395: The sentence is very long and difficult to understand.
The statement: "Since migrants and Australian-born workers have similar sensitivity to psychosocial job characteristics in terms of mental health" is very categorical and does not quite harmonise with the results, in my opinion.**

**Response:**

Thank you for this comment. We have revised this sentence now.

***The corresponding revision to the text is (Page 18, line 401-402):***

*Since we did not find statistically significant evidence that the impacts of psychosocial job characteristics on mental health were different between migrant and Australian-born workers……*

**R2-19. Lines 417-420: In what direction would common method bias probably affect the results? Usually this type of bias leads to stronger associations, which could be added.**

**Response:**

Thank you for this comment. We have revised this limitation.

***The corresponding revision to the text is (Page 20, line 431-432):***

*……thus common method bias could potentially inflate the relationship between psychosocial job characteristics and mental health.*

**R2-20. Lines 420-424: This could also make it difficult to observe differences between migrants from Non-English-speaking countries and English-speaking countries.**

**Response:**

Thank you for this comment. We have revised this limitation now.

***The corresponding revision to the text is (Page 20, line 434-436):***

*This exposure misclassification would make it harder to observe the differences in psychosocial job characteristic―mental health associations between migrants from Non-English-speaking countries and both native-born workers and migrants from Main-English-speaking countries.*

**R2-21. REFERENCES: Check Reference 49 (Laura).**

**Response:**

Thank you for this comment. We have deleted this reference.

**References**

1.	Landsbergis PA, Grzywacz JG, LaMontagne AD. Work organization, job insecurity, and occupational health disparities. Am J Ind Med. 2014;57(5):495-515. doi: 10.1002/ajim.22126. PubMed PMID: 23074099.

2.	Karasek RA. Job demands, job decision latitude, and mental strain: Implications for job redesign. Adm Sci Q. 1979;24(2):285-308. doi: Doi 10.2307/2392498. PubMed PMID: WOS:A1979GX41100007.

3.	Hayes AF. Introduction to mediation, moderation, and conditional process analysis: A regression-based approach. Second ed. New York: Guilford publications; 2018. 223-66 p.

4.	UCLA. How can i understand a categorical by continuous interaction? (Stata 12) | Stata FAQ 2020 [cited 2020 28 July]. Available from: <https://stats.idre.ucla.edu/stata/faq/how-can-i-understand-a-categorical-by-continuous-interaction-stata-12/>.

5.	Wege N, Dragano N, Erbel R, Jockel KH, Moebus S, Stang A, et al. When does work stress hurt? Testing the interaction with socioeconomic position in the Heinz Nixdorf Recall Study. J Epidemiol Community Health. 2008;62(4):338-41. doi: 10.1136/jech.2007.062315. PubMed PMID: 18339827.
