# Supplementary material for: Psychosocial job characteristics and mental health: Do associations differ by migrant status in an Australian working population sample?
Source: PLoS One. 2020 Nov 30;15(11):e0242906. doi: 10.1371/journal.pone.0242906 (PMC7703972; doi:10.1371/journal.pone.0242906)
Supplement: Attachment — Submitted filename: Response to Academic Editor.docx [file pone.0242906.s010.docx]

**Academic Editor**

We invite you to submit a revised version of the manuscript that addresses the points summarised by me. The revised manuscript will be reviewed by me rather than receiving another round of reviews. To be successful, the revision must include the following:

**AE-1: A more concrete answer to Reviewer 2's question in comment R2-3 "Is the performed LR tests the best way to assess effect measure modification?" comparing the various approaches available**

**Response:**

We have conducted our effect measure modification analyses using standard legitimate methods. Whether it is the “best way” is subject to debate—the key question is whether our approach is legitimate. Below, we outline some of the alternative methods, each of which has also been applied in our analyses, yielding—as expected—qualitatively identical results indicating a lack of statistical evidence for effect measure modification.

The fundamental approach is to fit a product term (exposure × effect modifier) and compare the model fit with versus without the product term in the model. Then “various approaches” available could refer to the choice of test statistics to compare the model fit. In Stata, the test statistic used for the likelihood ratio test (lrtest) is a Chi squared. Another approach applies a global F-test. Both tests provide similar p-value results, and a similar interpretation of the p-value can be made. Model fit can also be compared using Aikake’s Information Criteria (AIC) and Bayesian Information Criteria (BIC). These tests provide the same results again for our research question—no statistical evidence to indicate effect measure modification (these results have been provided in previous responses to review).

In many journals, a null result for comparative model fit testing is adequate in itself, and this is an adequate basis for ruling out effect measure modification in the case at hand and not presenting stratified results. Realising that we had two relatively small groups in our effect measure modification analyses (migrants resident ≤5 years [n=154] and 6-10 years [n=199]), we decided to present graphical results because the small sample sizes would limit statistical power and graphical results might suggest differences between groups (a clear demonstration of our awareness that ‘absence of [statistical] evidence is not evidence of absence’). This would be an important finding to inform future studies of the need for larger sample sizes. This is where the trouble with this paper began, because the slope for some results among migrants who had been resident for ≤5 years looked like they might be different from others, despite the null statistical results.

The ensuing debate with Reviewer 2 led us to Knol and VanderWeele (2012) and the ongoing debates in the literature on how best to present effect modification analysis results. This has been informative, but Knol and VanderWeele’s recommendations for presenting results on the relative magnitude and direction of differences in exposure-outcome relationships across effect modifier groups apply only to studies using binary or categorical outcome and exposure measures yielding Odds Ratios or Relative Risk measures (e.g., case-control studies). To achieve the same end of presenting the relative magnitude and direction of effect measure modification results in our paper, we have now added an analysis of differences in beta coefficients or slopes by each stratum of the relevant effect modifier, following on from a recommendation by Knol, Pestman and Grobbee (2011), cited by Reviewer 2, that “other methods to assess effect modification could be used, such as calculating a 95% confidence interval around the difference in effect estimates.” This was done in Stata using Pairwise comparisons of average marginal effects. We have revised the manuscript now to present these results in **Tables 3-5** (below). In summary, these analyses show that none of the differences in effect estimates between strata of any of the three effect measure modifiers tested were statistically significant: all of the 95% CIs comparing slope differences in **Tables 3-5** include zero. The figures which were previously presented in the main manuscript have now been shifted to Supporting Information and resized to increase readability (**S3-S5 Figs**, also included at the end of this document).

In summary, our effect measure modification analysis now includes 1) comparative model fit testing, 2) analysis of differences in effect estimates by strata of effect modifiers, and 3) descriptive presentation of graphical results. The first two analyses include formal statistical testing, and the third is for information (hence has been placed with Supporting Information). In this way, we provide both a global assessment of effect measure modification (analysis 1) as well as detail on the magnitude and direction of results within strata of effect modifiers (analyses 2 and 3).

***The corresponding revisions to the text are:***

***Methods***

*(Page 10-11, lines 228-240)*

*Third, effect measure modification of the job characteristic―mental health relationship by migrant status was tested by fitting product terms between psychosocial job characteristics and migrant status and comparing model fit between the model with versus without the product term using the Likelihood Ratio (LR) test. Considering that LR tests only provide measures of statistical significance without information on the relative magnitude and direction of differences in exposure-outcome relationships across effect modifier groups, comparative model fit testing was complemented for descriptive/explanatory purposes by graphing the relationships between psychosocial job characteristics and mental health by migrant status with 95% confidence intervals (CIs). In addition, the differences in the slope estimates of regression lines between effect modifier groups were assessed by average marginal effects comparisons [45]. LR test results or differences in slope estimates at p<0.05 was considered to constitute statistical evidence of effect measure modification and would justify the stratified presentation of job characteristic―mental health relationships as main findings.*

***Results***

*(Page 14, lines 299-300)*

*Likelihood ratio test results were uniformly null, providing no statistical evidence for any of our three measures of migrant status as effect modifiers of the job characteristic―mental health relationships……*

*(Page 14, lines 306-312)*

*When graphing effect measure modification results, it appeared that slopes for migrants who had been resident for ≤5 years might differ from other groups for both decision authority and skill discretion analyses (see* ***S3 and S4 Figs****; slope estimates are reported in* ***Tables 3 and 4****). We would also note the recently arrived migrant group (≤5 years resident) was the smallest of all migrant subgroups analysed (n=154,* ***Table 1****), and thus yielded the least precise beta/slope estimates. Consistent with the LR test results,* ***Tables 3 and 4*** *show that all of the 95% CIs comparing slope differences for skill discretion and decision authority analyses included zero.*

**Table 3. Relationships between skill discretion and mental health stratified by three measures of migrant status: the slope estimate within each stratum of migrant status and the differences in the slope estimates between groups, controlled for gender, age and educational attainment.**

|  | **Predicted mental health (MHI-5 score)** | | | | | |
| --- | --- | --- | --- | --- | --- | --- |
|  | **Migrant status measure one** | | **Migrant status measure two** | | **Migrant status measure three** | |
| **Slope estimates within the stratum of migrant status** | **Migrant status** | **Coef. (95% CI)** | **Migrant status** | **Coef. (95% CI)** | **Migrant status** | **Coef. (95% CI)** |
|  | Australian-born | 0.38 (0.24, 0.51)^***^ | Australian-born | 0.38 (0.24, 0.51)^***^ | Australian-born | 0.38 (0.24, 0.51)^***^ |
|  | Overseas-born | 0.48 (0.20, 0.75)^***^ | Main-ESC-born^†^ | 0.37 (-0.04, 0.78) | Arrived ≤5 years | 0.10 (-0.76, 0.95) |
|  |  |  | Non-ESC-born^†^ | 0.55 (0.19, 0.92)^**^ | Arrived 6-10 years | 0.59 (-0.16, 1.34) |
|  |  |  |  |  | Arrived ≥11 years | 0.51 (0.20, 0.83)^***^ |
| **Differences in slope estimates** | Overseas-born vs. Australian-born | 0.10 (-0.20, 0.41) | Main-ESC-born^†^ vs. Australian-born | -0.01 (-0.44, 0.43) | Arrived ≤5 years vs. Australian-born | -0.28 (-1.15, 0.58) |
|  |  |  | Non-ESC-born^†^ vs. Australian-born | 0.18 (-0.21, 0.57) | Arrived 6-10 years vs. Australian-born | 0.21 (-0.55, 0.98) |
|  |  |  | Non-ESC-born^†^ vs. Main-ESC-born^†^ | 0.18 (-0.37, 0.74) | Arrived ≥11 years vs. Australian-born | 0.13 (-0.21, 0.48) |
|  |  |  |  |  | Arrived 6-10 years vs. Arrived ≤5 years | 0.49 (-0.64, 1.63) |
|  |  |  |  |  | Arrived ≥11 years vs. Arrived ≤5 years | 0.42 (-0.49, 1.32) |
|  |  |  |  |  | Arrived ≥11 years vs. Arrived 6-10 years | -0.08 (-0.89, 0.74) |

Migrant status measure one: country of birth (COB), measure two: COB and English/Non-English dominant language of COB, measure three: COB and years since arrival; † Main-ESC-born: Born in a Main-English-speaking country; Non-ESC-born: Born in a Non-English-speaking country; Coef. was the beta coefficient; *** p<0.001, **p<0.01, *p<0.05.

**Table 4. Relationships between decision authority and mental health stratified by three measures of migrant status: the slope estimate within each stratum of migrant status and the differences in the slope estimates between groups, controlled for gender, age and educational attainment.**

|  | **Predicted mental health (MHI-5 score)** | | | | | |
| --- | --- | --- | --- | --- | --- | --- |
|  | **Migrant status measure one** | | **Migrant status measure two** | | **Migrant status measure three** | |
| **Slope estimates within the stratum of migrant status** | **Migrant status** | **Coef. (95% CI)** | **Migrant status** | **Coef. (95% CI)** | **Migrant status** | **Coef. (95% CI)** |
|  | Australian-born | 0.36 (0.28; 0.44)^***^ | Australian-born | 0.36 (0.28; 0.44)^***^ | Australian-born | 0.36 (0.28; 0.44)^***^ |
|  | Overseas-born | 0.37 (0.21; 0.54)^***^ | Main-ESC-born^†^ | 0.27 (0.03; 0.50)^*^ | Arrived ≤5 years | -0.04 (-0.73; 0.65) |
|  |  |  | Non-ESC-born^†^ | 0.47 (0.24; 0.70)^***^ | Arrived 6-10 years | 0.58 (0.08; 1.08)^*^ |
|  |  |  |  |  | Arrived ≥11 years | 0.37 (0.19; 0.55)^***^ |
| **Differences in slope estimates** | Overseas-born vs. Australian-born | 0.01 (-0.17; 0.19) | Main-ESC-born^†^ vs. Australian-born | -0.09 (-0.34; 0.15) | Arrived ≤5 years vs. Australian-born | -0.40 (-1.10; 0.29) |
|  |  |  | Non-ESC-born^†^ vs. Australian-born | 0.11 (-0.13; 0.36) | Arrived 6-10 years vs. Australian-born | 0.22 (-0.28; 0.73) |
|  |  |  | Non-ESC-born^†^ vs. Main-ESC-born^†^ | 0.21 (-0.12; 0.53) | Arrived ≥11 years vs. Australian-born | 0.01 (-0.18; 0.21) |
|  |  |  |  |  | Arrived 6-10 years vs. Arrived ≤5 years | 0.62 (-0.23; 1.48) |
|  |  |  |  |  | Arrived ≥11 years vs. Arrived ≤5 years | 0.42 (-0.30; 1.13) |
|  |  |  |  |  | Arrived ≥11 years vs. Arrived 6-10 years | -0.21 (-0.74; 0.32) |

Migrant status measure one: country of birth (COB), measure two: COB and English/Non-English dominant language of COB, measure three: COB and years since arrival; † Main-ESC-born: Born in a Main-English-speaking country; Non-ESC-born: Born in a Non-English-speaking country; Coef. was the beta coefficient; *** p<0.001, **p<0.01, *p<0.05.

*(Page 15, lines 328-332)*

*In the job insecurity―mental health relationships, the regression lines for all groups, including Australian-born workers and all migrant subgroups based on COB, dominant language of COB and years since arrival in Australia, were almost overlapping (see* ***S5 Fig****; slope estimates are reported in* ***Table 5****). Consistently,* ***Table 5*** *shows none of the differences in slope estimates between groups was significantly different from zero.*

**Table 5. Relationships between job insecurity and mental health stratified by three measures of migrant status: the slope estimate within each stratum of migrant status and the differences in the slope estimates between groups, controlled for gender, age and educational attainment.**

|  | **Predicted mental health (MHI-5 score)** | | | | | |
| --- | --- | --- | --- | --- | --- | --- |
|  | **Migrant status measure one** | | **Migrant status measure two** | | **Migrant status measure three** | |
| **Slope estimates within the stratum of migrant status** | **Migrant status** | **Coef. (95% CI)** | **Migrant status** | **Coef. (95% CI)** | **Migrant status** | **Coef. (95% CI)** |
|  | Australian-born | -1.08 (-1.18; -1.00)^***^ | Australian-born | -1.08 (-1.18; -1.00)^***^ | Australian-born | -1.08 (-1.18; -1.00)^***^ |
|  | Overseas-born | -1.03 (-1.21; -0.85)^***^ | Main-ESC-born^†^ | -0.99 (-1.26; -0.71)^***^ | Arrived ≤5 years | -0.79 (-1.44; -0.14)^*^ |
|  |  |  | Non-ESC-born^†^ | -1.07 (-1.31; -0.83)^***^ | Arrived 6-10 years | -1.27 (-1.80; -0.74)^***^ |
|  |  |  |  |  | Arrived ≥11 years | -1.03 (-1.24; -0.83)^***^ |
| **Differences in slope estimates** | Overseas-born vs. Australian-born | 0.05 (-0.15; 0.25) | Main-ESC-born^†^ vs. Australian-born | 0.10 (-0.19; 0.39) | Arrived ≤5 years vs. Australian-born | 0.29 (-0.37; 0.95) |
|  |  |  | Non-ESC-born^†^ vs. Australian-born | 0.01 (-0.25; 0.27) | Arrived 6-10 years vs. Australian-born | -0.18 (-0.72; 0.35) |
|  |  |  | Non-ESC-born^†^ vs. Main-ESC-born^†^ | -0.09 (-0.45; 0.28) | Arrived ≥11 years vs. Australian-born | 0.05 (-0.17; 0.27) |
|  |  |  |  |  | Arrived 6-10 years vs. Arrived ≤5 years | -0.48 (-1.32; 0.36) |
|  |  |  |  |  | Arrived ≥11 years vs. Arrived ≤5 years | -0.24 (-0.93; 0.44) |
|  |  |  |  |  | Arrived ≥11 years vs. Arrived 6-10 years | 0.23 (-0.33; 0.80) |

Migrant status measure one: country of birth (COB), measure two: COB and English/Non-English dominant language of COB, measure three: COB and years since arrival; † Main-ESC-born: Born in a Main-English-speaking country; Non-ESC-born: Born in a Non-English-speaking country; Coef. was the beta coefficient; *** p<0.001, **p<0.01, *p<0.05.

*(Page 16, lines 341-345)*

*In summary, there was no statistical evidence supporting any of our three measures of migrant status as effect modifiers of the relationships between psychosocial job characteristics and mental health,* *despite the suggestion of some differences by visual inspection of graphical results. The main finding of our effect measure modification analysis is that the relationship between the three job characteristics and mental health does not differ by any of the three measures of migrant status used.*

***Discussion***

*(Page 18, lines 380-383)*

*We did, however, find suggestive evidence that skill discretion and decision authority may not be associated with the mental health of most recently arrived migrants, suggesting associations for these two psychosocial job characteristics are stronger in Australian-born workers (see* ***Tables 3 and 4*** *and* ***S3 and S4 Figs****);……*

**AE-2: The implementation of the recommendations by Knol and VanderWeele (2012) were relevant; if at all, it should be further outlined why recommendations were not followed.**

**Response:**

This has now been answered above in our response to AE-1. To summarise:

The specific recommendations for presenting relative excess risk due to interaction (RERI) in effect modification results by Knol and VanderWeele (2012) apply only to situations where the outcome, exposure, and effect modifier are binary or categorical variables (e.g., case-control studies). In our paper, we are relating continuous measures of outcome and exposure, and assessing that relationship for effect measure modification by binary and categorical measures of migrant status.

To provide detail on the relative magnitude and direction of effect measure modification results in our paper (the purpose of Knol & VanderWeele’s recommendations to provide RERI or Synergy Index results for binary or categorical measures), we have assessed differences in effect estimates with 95% CIs across strata of effect modifiers (Knol, Pestman & Grobbee 2011), using pairwise comparisons of average marginal effects, as detailed in our preceding response.

**AE-3: A more careful application of the principle "Absence of evidence is not the same as evidence of absence" (as suggested by Reviewer 2).**

**Response:**

We believe the reviewer and we were sharing the same recognition that there may be effect measure modification that our study was underpowered to detect. A non-critical acceptance of our comparative model fit testing results would have precluded presentation of graphical effect modification results. This clearly demonstrates that we believe that “absence of evidence is not the same as evidence of absence.” We hope this is now clear.

**AE-4: A revised first sentence of the abstract.**

**Response:**

We have revised this sentence.

***The corresponding revision to the text is (page 2, line 32-33):***

*Migrant workers may experience higher burdens of occupational injury and illness compared to native-born workers,……*

**AE-5: A revision of the following anthropomorphism in line 202: “8983 observations had completed data”.**

**Response:**

Thank you for this comment. We have revised this sentence.

***The corresponding revision to the text is (page 9, line 201-202):***

*Among these 10569,* *8983 observations had complete items for the three psychosocial job characteristic scales,……*

**AE-6: Table S1 in the manuscript (Reviewer’s comment: S1 Table is important, as it shows why there is effect modification for newly arrived migrants; their mean scores for psychosocial factors and mental health differ from the other migrant groups and Australian-born people).**

**Response:**

**S1 Table** provides univariate descriptive information (distributions, summary measures) on psychosocial factors and MHI-5 scores for Australian-born workers and each migrant subgroup, but nothing on exposure-outcome associations. Nothing about the associations between job characteristics and mental health, nor difference in those associations by migrant status can be concluded from this table.

**Reference**

Knol, MJ, Pestman, WR & Grobbee, DE 2011, 'The (mis) use of overlap of confidence intervals to assess effect modification', European Journal of Epidemiology, vol. 26, no. 4, pp. 253-4.

Knol, MJ & VanderWeele, TJ 2012, 'Recommendations for presenting analyses of effect modification and interaction', International Journal of Epidemiology, vol. 41, no. 2, pp. 514-20.

**S3 Fig. Relationships between skill discretion and mental health stratified by three measures of migrant status.**

Graphs generated from linear regressions with the product terms of skill discretion and migrant status and controlled for gender, age and educational attainment. Mental health was measured by the MHI-5 score. Shown with 95% CIs.

**S4 Fig. Relationships between decision authority and mental health stratified by three measures of migrant status.**

Graphs generated from linear regressions with the product terms of decision authority and migrant status and controlled for gender, age and educational attainment. Mental health was measured by the MHI-5 score. Shown with 95% CIs.

**S5 Fig. Relationships between job insecurity and mental health stratified by three measures of migrant status.**

Graphs generated from linear regressions with the product terms of job insecurity and migrant status and controlled for gender, age and educational attainment. Mental health was measured by the MHI-5 score. Shown with 95% CIs.
